# Polygenic transcriptome risk scores (PTRS) can improve portability of polygenic risk scores across ancestries

**DOI:** 10.1186/s13059-021-02591-w

**Published:** 2022-01-13

**Authors:** Yanyu Liang, Milton Pividori, Ani Manichaikul, Abraham A. Palmer, Nancy J. Cox, Heather E. Wheeler, Hae Kyung Im

**Affiliations:** 1 Section of Genetic Medicine, Department of Medicine, The University of Chicago, Chicago, IL USA; 2 Department of Genetics, Perelman School of Medicine, University of Pennsylvania, Philadelphia, 19104 PA USA; 3 Center for Public Health Genomics, University of Virginia, Charlottesville, VA USA; 4 Department of Psychiatry, University of California San Diego, San Diego, CA USA; 5 Institute for Genomic Medicine, University of California San Diego, San Diego, CA USA; 6 Vanderbilt Genetic Institute, Vanderbilt University Medical Center, Nashville, TN USA; 7 Department of Biology, Loyola University Chicago, Chicago, IL USA; 8 Program in Bioinformatics, Loyola University Chicago, Chicago, IL USA; 9 Department of Public Health Sciences, Stritch School of Medicine, Loyola University Chicago, Maywood, IL USA

## Abstract

**Background:**

Polygenic risk scores (PRS) are valuable to translate the results of genome-wide association studies (GWAS) into clinical practice. To date, most GWAS have been based on individuals of European-ancestry leading to poor performance in populations of non-European ancestry.

**Results:**

We introduce the polygenic transcriptome risk score (PTRS), which is based on predicted transcript levels (rather than SNPs), and explore the portability of PTRS across populations using UK Biobank data.

**Conclusions:**

We show that PTRS has a significantly higher portability (Wilcoxon *p*=0.013) in the African-descent samples where the loss of performance is most acute with better performance than PRS when used in combination.

**Supplementary Information:**

The online version contains supplementary material available at (10.1186/s13059-021-02591-w).

## Background

Polygenic risk scores (PRS) for a variety of traits are promising to become accurate enough to be useful for clinical practice and realize the longstanding goal of personalized medicine. PRS for coronary artery disease (CAD) have been shown to provide prediction that has been compared to monogenic mutations of hypercholesterolemia [1]. In practice, PRS may impact a larger proportion of patients compared to monogenic mutations; for example, PRS for CAD provide potentially actionable information for 8% of the population (for whom the risk increases by three-fold) whereas known monogenic mutations are only informative for about 0.4% of patients. However, a major limitation of this approach is that PRS developed in one human ancestry group do not perform well in other ancestry groups, limiting their utility and exacerbating already severe health disparities [2, 3]. This problem is being addressed by large efforts such as Human Heredity and Health in Africa (H3Africa) [4], Million Veterans Project [5], AllofUs [6], and TOPMED [7] that are recruiting individuals from diverse ancestry groups.

However, these efforts are time consuming, enormously expensive and will have to be repeated at scale, for numerous traits, across numerous ancestry groups. Therefore, methods that can use GWAS studies from one population for prediction in other ancestry groups are highly desirable. Analysis of GWAS conducted in different populations suggested that a considerable fraction of causal SNPs are shared across populations [8]. Hence, efforts to develop methods that transfers knowledge about the influence of genes on traits across populations could help improve prediction in underrepresented ancestry groups in a cost-effective manner.

It is now widely understood that many association signals are driven by their effects on the transcriptome. PrediXcan [9] and other TWAS methods [10, 11] leverage reference transcriptome datasets to train prediction models of gene expression levels and correlate the genetically predicted gene expression levels with complex traits to identify causal genes. Assuming that the role of genes on traits is conserved across populations, we hypothesized that prediction at the level of estimated transcript abundance rather than SNPs might help the portability across populations.

Therefore, we propose the polygenic transcriptomic risk score (PTRS) as a gene-based complement to the PRS that can help improve portability across human ancestry groups. We recognize that PTRS alone does not outperform the state of the art PRS [12]. However, integrating PTRS to PRS construction has many desirable properties. One advantage of PTRS is that the smaller number of features (tens of thousands of genes rather than millions of SNPs); it means that optimizing the parameters to build PTRS is more manageable than PRS. Another advantage of PTRS is that training transcriptome prediction models requires much smaller samples than training PRS and can then be used for prediction of many different traits. Furthermore, training data for non-European-descent individuals are becoming increasingly available. Finally, because PTRS is gene-based, it is inherently more biologically interpretable than PRS.

In this paper, we explore the properties of PTRS using the UK Biobank (UKB), which provides genotype and phenotype data in half a million individuals [13]. Although the majority of participants in UKB are of European-descent, several thousand individuals of non-European descent are also available and can be used to compare prediction by PRS and PTRS across ancestries. We start by quantifying how much of the trait heritability can be explained by the genetically predicted transcriptome. We then build PRS and PTRS for a range of complex traits and compared their prediction accuracy and portability across populations.

## Results

Before describing the results, we define and clarify some terminology. In this paper, there are two types of prediction: (1) gene expression level prediction from genotype data and (2) complex trait prediction using PRS or PTRS. PRS uses genotype data directly and PTRS uses linear combinations of genotypes representing predicted gene expression levels. To simplify exposition, we will only use the term *training* for the calculation of weights for predicting gene expression levels using genotype data. The *training* of transcriptome (gene expression levels) prediction weights had been performed previously, and we simply downloaded them from predictdb.org. When we estimate optimal weights for PRS and PTRS, we will use the terms *building* or *constructing*. We performed the *building* of PRS and PTRS using the *discovery* set. The testing of the risk scores, PRS and PTRS, were performed in what we call the *target* sets. For the remainder of the paper, we will refer to individuals by their ancestry and drop the -descent suffix. Unless otherwise clarified, we will use the term transcriptome to mean the set of predicted expression levels of genes. GTEx EUR transcriptome should be interpreted as the set of predicted gene expression levels using weights trained in European-descent samples from GTEx. Similarly, MESA EUR transcriptome, will refer to the predicted transcriptome using weights trained with the MESA European-descent samples. MESA AFHI transcriptome will refer to the predicted transcriptome using weights trained with a combination of African American-descent and Hispanic individuals from the MESA study.

***Experimental setup***

**Fig. 1:**
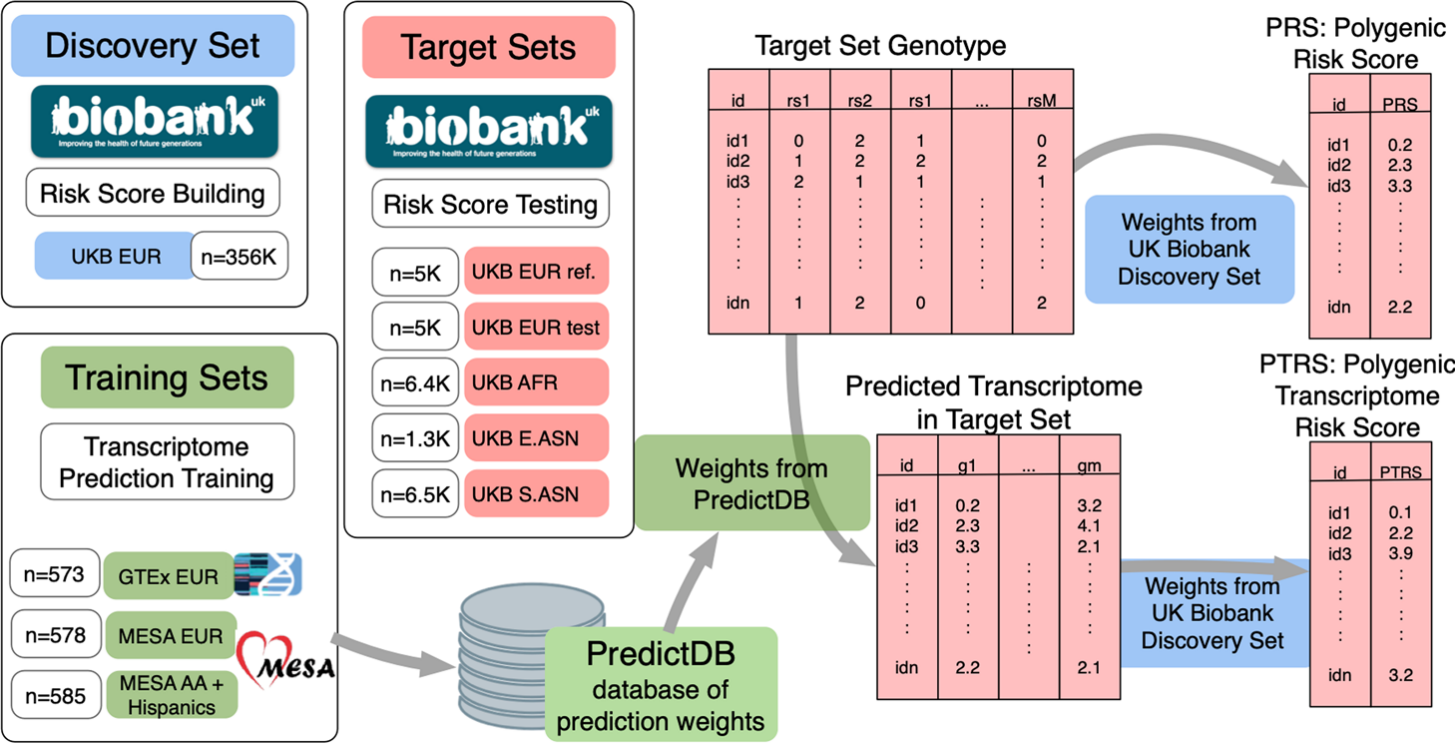
Experiment setup. Summary of the experimental set up used for testing the portability of PRS and PTRS across populations. The weights for calculating PRS and PTRS were estimated in the *discovery set*, which consisted of 345K randomly sampled individuals of European-descent from the UK Biobank. The *training* sets where the weights for the prediction of transcriptomes were computed are shown in green. We downloaded the weights trained previously from predictdb.org. We sampled 5 *target sets* from the UK Biobank for testing the risk scores: two randomly sampled sets of European-, one African-, one East Asian-, and one South Asian-descent individuals. For each of the 5 *target* sets, predicted transcriptomes were calculated using the weights trained in each of the three *training* sets: GTEx EUR, MESA-EUR, and MESA-AFHI

An overview of the experimental setup describing the discovery, training, and target sets used in the paper is shown in Fig. 1. We randomly selected 356,476 unrelated Europeans in the UK Biobank for the discovery set. For testing the performance of risk scores, we constructed 5 target sets with participants of various ancestries in the UK Biobank. We used 6,413 African-, 1,326 East Asian-, and 6,479 South Asian- descent individuals for the non European target sets. We also reserved two randomly selected sets of 5000 Europeans as additional target sets. One was selected as the EUR reference set and the second European target was used as a test set to assess the variability of the results within the same ancestry.

For predicting the transcriptome, we downloaded prediction weights from multiple ancestries collected in predictdb.org. The first set of models had been trained in European individuals from the GTEx v8 release [14] in whole blood. The second set of models had been trained using array-based expression in monocyte samples of Europeans, African Americans, and Hispanics from the MESA cohort [15].

For our tests, we focused on the 17 anthropomorphic and blood phenotypes used by Martin et al. [3]. We sampled our discovery and target sets randomly so that there is no exact match with Martin et al.’s discovery set.

***Predicted transcriptome captures a higher portion of chip heritability than expected***

Before building PTRS, we started by determining whether prediction of traits was possible using just predicted transcriptomes. If feasible, we also wanted to know how much of the trait variation can be captured by the predicted transcriptome. For this purpose, we calculated the proportion of variance explained (PVE) by the predicted transcriptome assuming random effects of gene expression levels. The approach is analogous to standard SNP-heritability estimation [16]. For heritability estimation, one would use the genetic relatedness matrix. Here, we use the “predicted expression relatedness matrix.”

In this section, we calculated the predicted transcriptome using the GTEx EUR weights using the European target set genotype data. Using these predicted expression levels, we calculated the “predicted expression relatedness matrix” (instead of the genetic relatedness matrix) and applied the standard restricted maximum likelihood estimation to calculate the proportion of variance explained by the predicted transcriptome.

Since the PVE for each trait will depend on the heritability of the trait, we calculated the proportion of PVE divided by the heritability of the trait. This quantity represents the proportion of heritability explained by the predicted transcriptome. We also estimated the SNP-heritability using the restricted maximum likelihood approach in the same cohort.

**Fig. 2:**
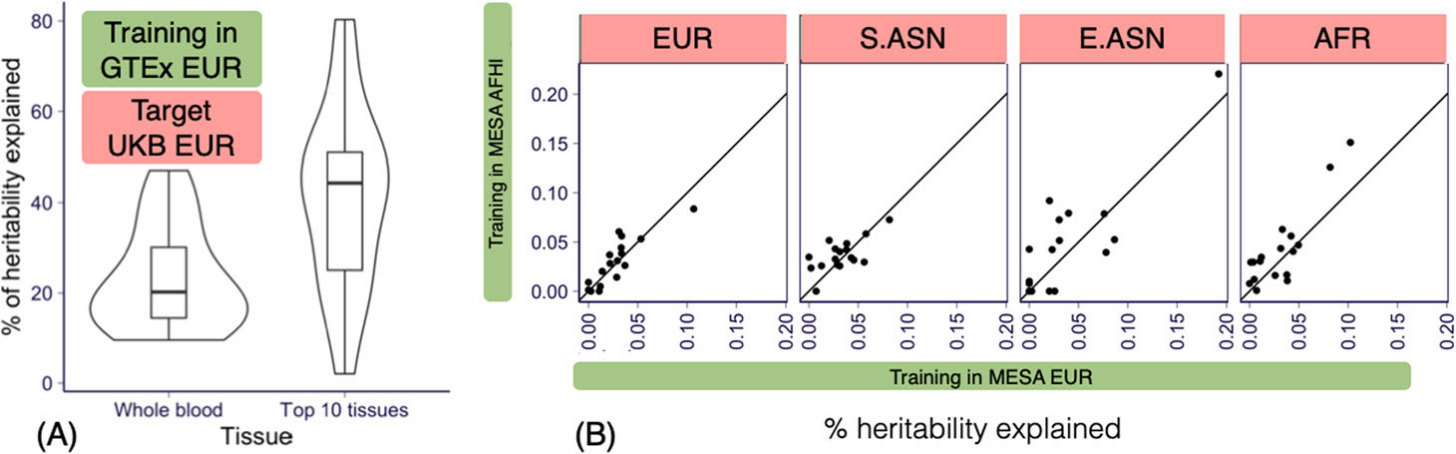
Proportion of variance explained (PVE) by the predicted transcriptome. **a** The ratio of PVE (the proportion of phenotypic variation explained by the predicted transcriptome) of GTEx EUR transcriptome model over the chip heritability using whole blood on the left and using 10 tissues on the right. The 10 tissues with highest sample sizes were selected from GTEx (muscle, adipose, tibial artery, breast, lung, fibroblast, lung, tibial nerve, and skin, with sample sizes ranging from 337 to 602). Notice that in general we used GTEx whole blood and MESA monocyte-based predictors except for this panel where we used 10 tissues from GTEx. **b** The PVE of MESA EUR-based predicted transcriptome (x-axis) and MESA AFHI-based predicted transcriptome (*y*-axis) are shown. Each panel presents the results calculated in one ancestry group and each point presents one trait

Figure 2a shows the distribution of the proportion of heritability explained by the GTEx EUR transcriptome calculated in the EUR target set. We found that the GTEx EUR whole blood based predicted transcriptome captured on average 22.9% (s.e.=2.9%) of the trait heritability. Chip heritability and PVE values can be found in Additional file 2: Table S4 and Additional file 3: Table S5. This result is largely consistent to the estimates reported previously by [17] for the subset of traits used here which were mostly blood related. Notice that the predicted transcriptome had fewer than 1% of the number of features used in the calculation of the chip heritability (fewer than 10K genes predicted in whole blood compared to roughly 1 million independent SNPs). Therefore, this result constitutes a 20-fold increase in the per feature proportion of heritability explained.

***Aggregating predicted transcriptomes in multiple tissues increases the PVE***

To explore ways to increase the proportion of variance explained (PVE), we calculated the proportion explained collectively by the transcriptome predicted in 10 tissues selected among the ones with largest sample sizes in GTEx, including muscle, adipose, tibial artery, breast, lung, fibroblast, lung, tibial nerve, and skin, with sample sizes ranging from 337 to 602 (Additional file 1: Table S3). As anticipated, we found that, collectively, the predicted transcriptomes in 10 tissues explained a larger portion of heritability: on average, 35.5% (s.e. = 4.7%) of the heritability corresponding to a 48% increase relative to whole blood alone. This result suggests that adding transcriptomes from multiple tissues will improve predictions in general.

***Matching training and target ancestries may increase the proportion of variance explained***

So far, we have used GTEx EUR transcriptomes to calculate the PVE. It is reasonable to assume that matching the training and target populations, i.e., using transcriptomes trained in the same population as the target sets should be beneficial. We tested this hypothesis here.

We took advantage of the availability of trans-ancestry transcriptome prediction models from the MESA cohort [15]. One of them (MESA-EUR) was trained in a European population and the other one (MESA AFHI) was trained in a combination of African American and Hispanic populations (Additional file 1: Table S3). We decided to use the combined (African American and Hispanic) transcriptome prediction since the similarity of the sample sizes (578 vs 585) would make the comparison with the European trained models more fair.

We found that (Fig. 2b) in the African target set, using the ancestry matched MESA AFHI transcriptome yielded a higher PVE albeit not significant (1.1%, *p*=0.065) proportion of variance explained than when using the MESA EUR transcriptome. For the European target set, the difference between using the MESA AFHI or the EUR transcriptomes was close to 0 (0.3%, *p*=0.50). This lack of significance could be attributed to the limitations of the array-based MESA transcriptome data prompting the need to generate improved non-European transcriptome predictors.

***Building PRS and PTRS***

After having determined that it is possible to capture a significant portion of trait variability using predicted transcriptome, we proceeded to build the PRS and PTRS in our discovery set (356K Europeans from the UK Biobank).

We built PTRS weights using elastic net, a regularized linear regression approach, which selects a sparse set of predicted expression features to make up the PTRS. For PRS weights, we used the standard LD clumping and *p*-value thresholding approach (see details in the “Methods” section). To rule out that using elastic net for PTRS instead of clumping and thresholding as done for PRS was driving our conclusions, we also ran the comparison using clumping and thresholding for PTRS and found no change in the substance of our results (Additional file 1: Figures S1 and S2).

We quantified the prediction accuracy in each target set using the partial *R*^2^ ($\widetilde {R}^{2}$), which provides a measure of correlation between predicted and observed outcomes with the added benefit of taking covariates into account (see details in the “Methods” section). We split the target set into validation and test set and determined the hyperparameters (penalty for elastic net PTRS and *p*-value threshold for PRS/PTRS) in the validation set and calculated the $\widetilde {R}^{2}$ under the selected hyperparameter in the test set. To reduce the stochasticity of the estimated performance, we repeated the random splitting 10 times and report the average $\widetilde {R}^{2}$ across the ten splitting schemes. The weights were calculated in the discovery set for the different hyperparameters.

***PTRS’ prediction accuracy approaches the approximate upper bound estimated by their PVE***

**Fig. 3:**
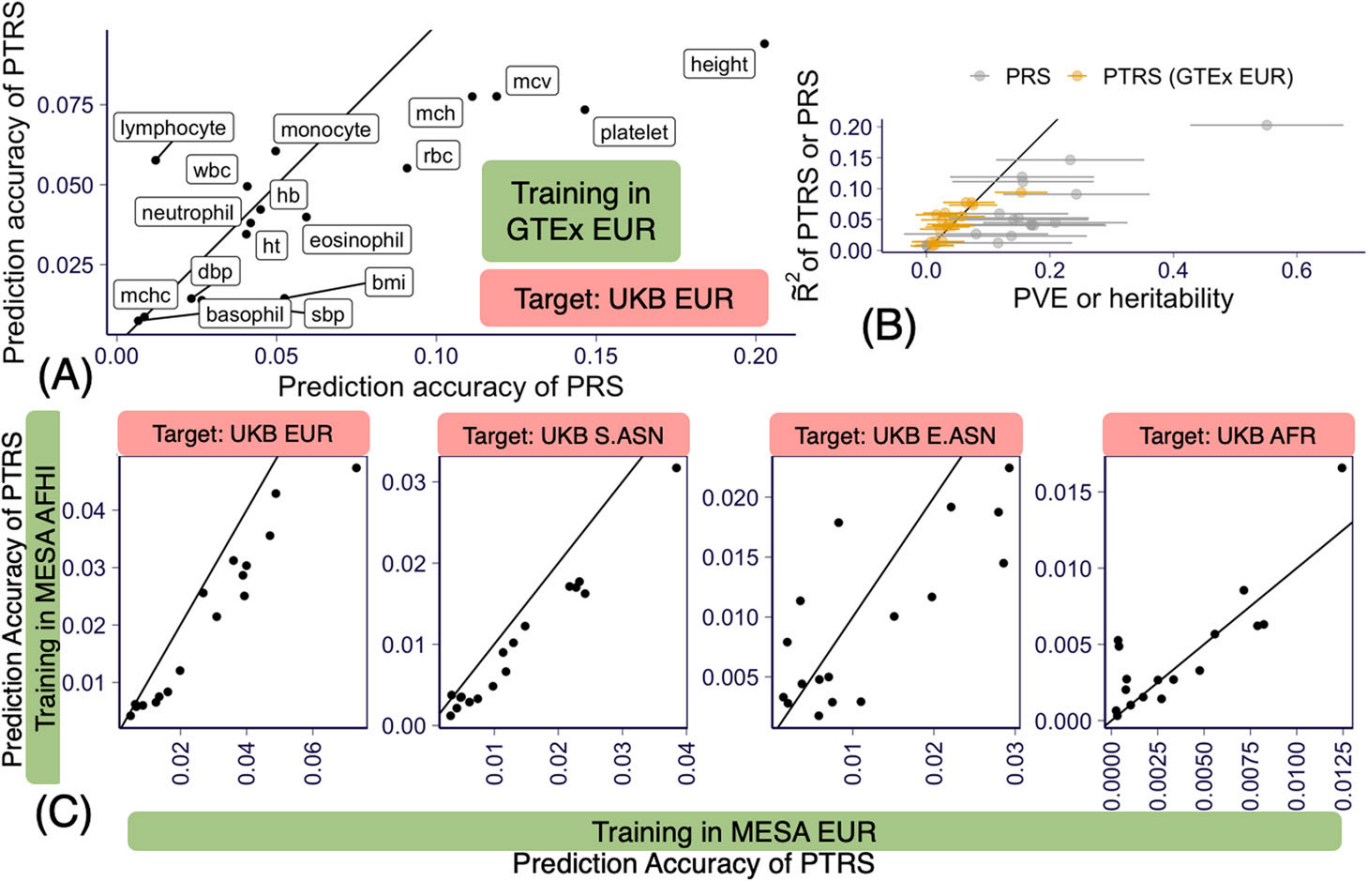
Prediction accuracy of predicted transcriptome risk scores (PTRS). **a** Prediction accuracy, measured by partial $\widetilde {R}^{2}$, of PTRS (on *y*-axis) compared to the accuracy of PRS (on *x*-axis). **b** Prediction performance is shown on *x*-axis and heritability (for PRS) and proportion of variance explained (for PTRS) are shown on *y*-axis. **c** Prediction accuracy of MESA AFHI PTRS vs MESA EUR PTRS in the 4 target populations

The prediction accuracy of PTRS (GTEx EUR based) was on average lower than the accuracy of PRS (paired *t*-test *p* = 0.03) as shown for the 17 traits in Fig. 3a for the European target set and in Additional file 1: Fig. S3 for the other ancestries. The prediction accuracy values for PRS and PTRS are listed in Additional file 4: Table S6 and Additional file 5: Table S7. Note that since predicted transcriptomes were explaining about a fifth of the heritability (i.e., what common genetic variants could explain), we would expect that the prediction performance of PTRS would be about a fifth of the PRS performance. However, PTRS performance was around half of the PRS performance. This better than expected performance (50% relative to PRS instead of the expected 20%) suggests that integrating predicted transcriptomes and other omics is a promising avenue to improve PRS performance in general.

We found that PTRS was much closer to the optimal performance upper bound (estimated as the PVE) than PRS was to its upper bound (heritability), as shown in Fig. 3b, where each score is compared to its upper bound. This is consistent with the reported monotonically increasing relationship between a measure of prediction performance (correlation between the genetic component and the predicted values, slightly different from our definition, using lowercase *r* for distinction) and the per-feature heritability/PVE: $r^{2} = \frac {n \cdot h^{2}/n_{\text {features}}}{ 1 + n \cdot h^{2}/n_{\text {features}}}$ [18]. The higher than expected performance of PTRS indicates that the predicted expression of each gene carries a higher per feature PVE than genetic variant’s per feature heritability.

***Matching training and target ancestries may improve prediction accuracy***

We have suggestive evidence that matching training and target ancestries can improve the PVE in the African population. To test whether matching the training and target ancestries would improve the PTRS prediction accuracy, we examined the difference between using the African transcriptome (MESA AFHI) vs the European transcriptome (MESA EUR).

For the European target set, the European transcriptome based PTRS had better accuracy than the AFHI transcriptome based one, with an gain of 0.74% (s.e.=0.096%) when using European vs AFHI, as hypothesized. For the African target set, however, the difference was not significant indicating that improvements in prediction across ancestries are probably needed to detect the potential gain.

To avoid differences due to having different number of predicted genes, PTRS were built using only the genes that were present in both training sets, EUR and AFHI (see details in the “Methods” section).

***PTRS improves portability into the African-descent population***

To test our hypothesis that PTRS can generalize more robustly across populations than the standard PRS, we defined “*portability*” as the predictive accuracy in each population relative to the European reference target set (EUR ref.). This is calculated as the ratio of the $\widetilde {R}^{2}$ in the target population divided by the $\widetilde {R}^{2}$ in the European reference target set. Thus, by definition the portability in the European reference set is 1.

**Fig. 4:**
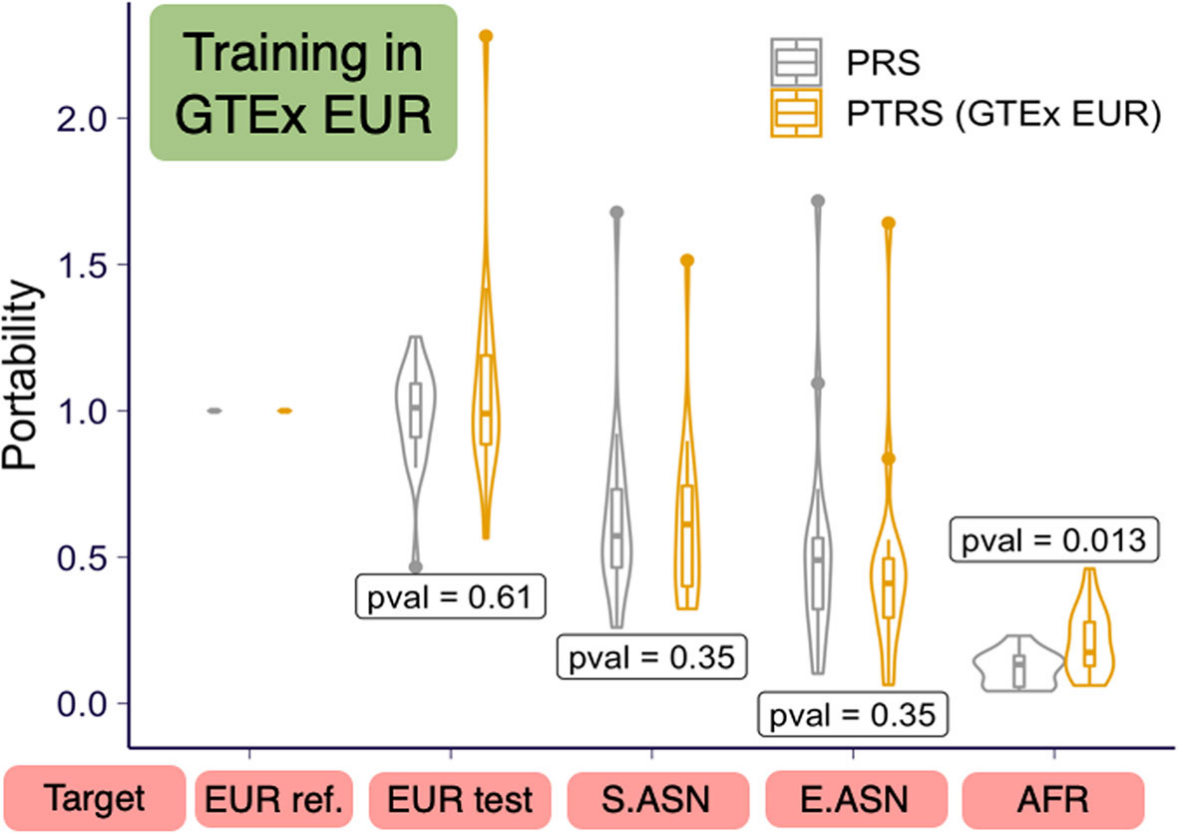
Portability of PTRS for 17 quantitative phenotypes in UK Biobank. The portability of PTRS trained and calculated using GTEx EUR whole blood samples are shown in yellow with the PRS shown in gray. “EUR ref.” set is used as the reference population in the calculation of portability (see details in the “Methods” section) so that the portability is always 1. Recall that the absolute performance of PTRS is, on average, lower than PRS as shown in Fig. 3a and Additional file 1: Figure S3

Consistent with reports by [3], the portability of PRS degrades with the genetic distance to the European discovery set as shown in gray in Fig. 4a. The portability of PTRS (shown in orange) also decreases with genetic distance to the discovery set, with the African target sets showing the largest loss of accuracy, as expected. However, we also observed that the portability of PTRS in the African target set was significantly higher than the portability of PRS (Wilcoxon *p*=0.013). These results provide strong proof of principle that integrating predicted transcriptome as done with PTRS has the potential to improve portability of risk scores across populations despite the limitations of currently available AFR training data. In the European test set, we observed quite a bit of variability in the portability, ranging from 0.47 to 2.28, despite the fact that both European target sets were randomly sampled from the same European UK Biobank participant set. As expected, the median portability in the second EUR target set is centered around 1.

We verified that the increase in portability of PTRS in the African target set was not driven by the use of elastic net instead of clumping and thresholding for building the PTRS as shown in Additional file 1: Figure S2 where portability is shown for the clumping and thresholding approach. By looking at the comparison between prediction performance and portability (Additional file 1: Figure S4), we found no evidence that the increase in portability in the African target set could be driven by low prediction performance in the set.

Furthermore, we examined whether the population-matched transcriptome models could improve portability using the MESA models. However, we did not observe significant improvement relative to PRS (Additional file 1: Figure S5), which could be due to the lower quality of the prediction based on the older array technology.

***Combining PTRS and PRS can improve performance and portability over PRS alone***

**Fig. 5:**
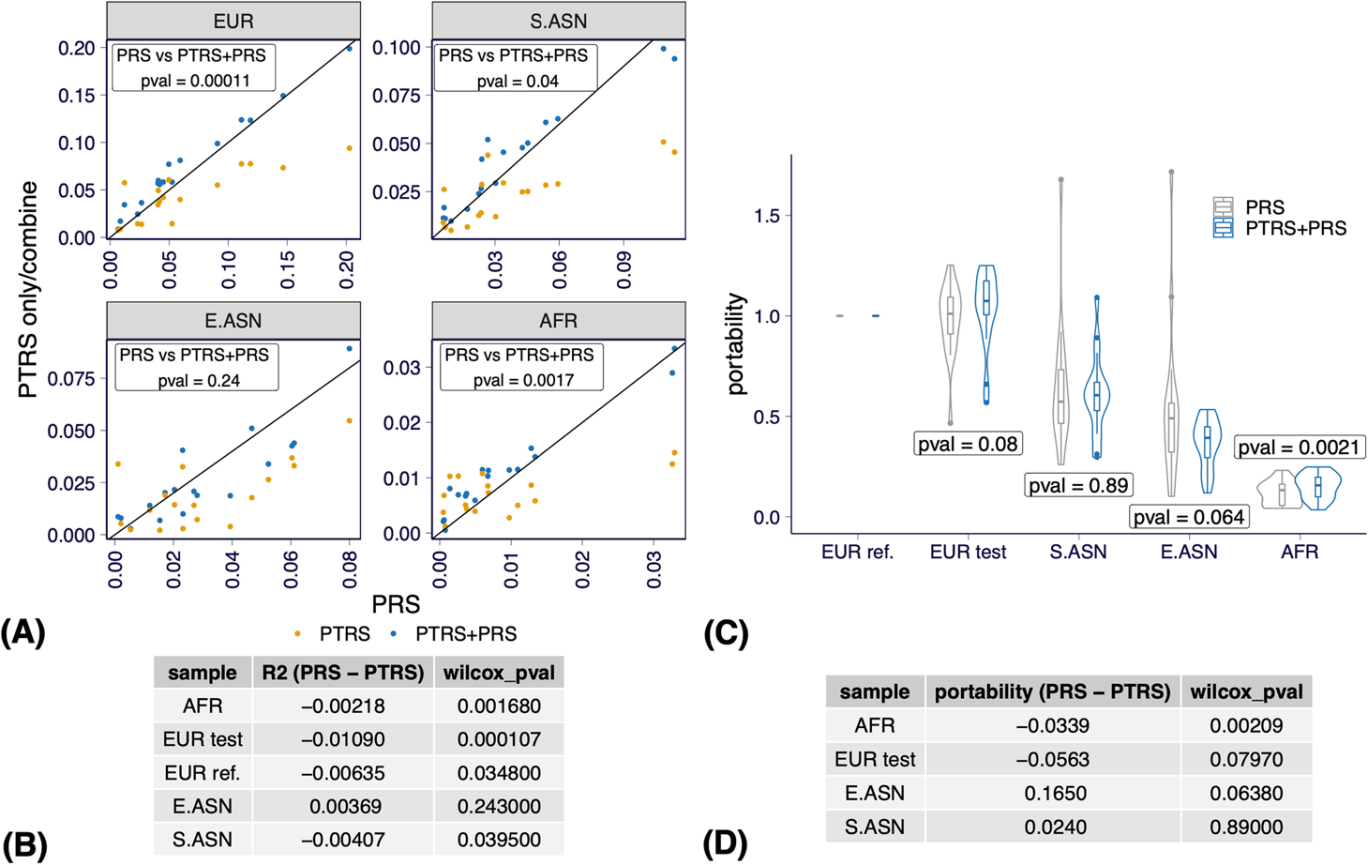
Prediction performance and portability of the combined score PTRS*=PTRS+PRS vs PRS. **a** The prediction accuracy of the PRS (*x*-axis) is compared against the prediction accuracy of the PTRS (yellow) or the combined score (blue) on *y*-axis. The results on all of the 17 quantitative traits are shown. Each panel corresponds to one ancestry group. The *p*-values of the difference in prediction performance were calculated using a paired Wilcoxon signed rank test. **b** A summary of the difference between the prediction accuracy of PRS and the combined score (PTRS*=PRS+PTRS) is shown for each of the ancestry group. The second column shows the mean difference and the third column shows the *p*-values (paired Wilcoxon signed rank test comparing). **c** The portability of PRS (gray) and the combined score (blue) is shown for each ancestry group. The violin and box plots summarize the results from the 17 quantitative traits. Paired Wilcoxon signed rank test *p*-values are shown. **d** A summary of the difference between the portability of PRS and the combined score is shown for each of the ancestry group. The second column shows the mean difference and the third column shows the results of the paired Wilcoxon signed rank test comparing the portability of PRS versus the combined score

To demonstrate the benefits of combining PTRS and PRS, we tested the prediction performance and portability of a weighted sum of PRS and PTRS. See description of the calibration of the weights in the “Methods” section. Combining the elastic net-based PTRS and the clumping and thresholding-based PRS, the combined score yielded significantly higher performance than PRS alone in all but E.ASN ancestry (Fig. 5a and 5b). We also found significantly better portability of the combined score compare to PRS alone in the African target set (Fig. 5c and 5d).

To rule out that the improvement in performance was due to the better prediction approach used for PTRS, we also performed the previous analysis using clumping and thresholding PTRS (see the “Methods” section) and found the sign of the gain remained unchanged although the differences were no longer significant. The gain in portability in the AFR population remained significant (*p*=0.011). (Additional file 1: Figures S6 and S7)

Taken together, our results provide support to our hypothesis that PTRS can improve the portability of PRS in general. Also, they suggests that adding transcriptomes predicted in other tissues and other omics data are promising avenues to further improve PRS portability.

## Discussion

In this paper, we introduced the polygenic transcriptomic risk score and used it to address a major problem in human genetics, namely the poor ability to use genotype to predict phenotype using PRS that are trained in one ancestry group but applied to another ancestry group. We started by establishing that prediction of complex traits using the predicted transcriptome is possible by showing that the total trait variation explained via predicted transcriptomes ranges from 22.9% (using whole blood) to 35.5% (with a broader sets of tissues) of the SNP heritability, i.e., the total variation that can be explained using common SNPs. Promisingly, the actual predictors built on predicted transcriptomes had performances that were more than double the expected 22.9% of the PRS performance. We found that the portability of PTRS was significantly higher than the portability of PRS in the African target set, which is important since the African populations are the most affected by the Eurocentric bias in GWAS studies [3]. Our study results suggest that investing in multi-omic studies of diverse populations may be an effective way to reduce current genomic disparities by taking better advantage of existing GWAS studies. The increased performance of the combined score PRS+PTRS over PRS further supports this idea.

In this paper, we have explored the use of PTRS to address the limited portability of PRS across ancestry groups. One intriguing application of our PTRS method is to transfer the polygenic knowledge derived in humans to other model systems. Currently, most attempts to follow up on human GWAS findings in model organisms focus on individual genes, despite the overwhelming evidence that individual genes seldom contribute even 1% of trait variability. As we improve our PTRS, it would be possible to build models based on human discovery sets and then use them to predict those traits in model organisms based on either measured or predicted transcript levels. Whether this approach could successfully predict traits that can be measured in other species (e.g., body size, blood pressure) is currently unknown. The success of PTRS for cross-species translation is theoretically dependent on the extent to which gene expression differences have similar impacts across species. However, if we could build a transferable PTRS, we could envision to run experiments on model organisms, measure their transcriptomes (in the right context and tissue), and predict complex traits (e.g. behavioral traits) that are hard or impossible to measure. This platform could be used to test various interventions and obtain effects on phenotypes that are more relevant to humans, opening up completely novel lines of investigation.

Our study points to promising strategies to improve risk prediction in general but it also has several limitations. First, PTRS are based on prediction models of gene expression traits which we estimated to account for less than a third of the total variability in the complex traits considered here. We expect this limitation to be mitigated as additional transcriptome reference sets in different contexts as well as other omics data covering mediating mechanisms missed in current models. The increase in the proportion of variance explained from a fifth when using whole blood predictors alone compared to over 35.5% when using 10 tissues indicates that much can be gained by increasing the breadth of reference omic data. Second, we used single tissue prediction models for most of the analysis in this paper, which captured a fifth of the variation in the complex traits here. This can be improved by developing approaches to integrate multiple tissue models. Third, weights for PRS were calculated using GWAS summary results (thresholding and pruning method) whereas PTRS weights were calculated using individual level data due to computational considerations. Individual-level based PRS would likely perform better, although whether they would be more portable is not obvious. For reassurance, we have ruled out the possibility that the portability improvement in the African target set was due to this different treatment of PTRS and PRS by re-calculating the portability of PTRS using the same approach as PRS (summary statistics based clumping followed by *p*-value thresholding). Fourth, higher quality prediction models of the transcriptome in non-European ancestries are limited. Here, we used predictors trained in monocyte samples assayed with older array technology. Multiple ancestry models are currently being generated by us and other groups. For example, the MESA TOPMED project has assayed RNAseq, protein, methylation, and metabolomics data in African Americans, Hispanics, and Asian ancestry individuals which will allow the development of improved prediction models. Currently, our methods are based on transcriptomic data from bulk tissues; however, just as the choice of tissue is important, the choice of tissue and cell type has the potential to provide even better performance; however, the availability of such data, as well as the methods for implementing it, are currently limited. Furthermore, prediction models are currently based on variants in the vicinity of the gene. Improved predictors with genome-wide variants should be investigated. Finally, other approaches such as prioritizing functional annotations for variant selection [19], more diverse set of variants [20], and better leveraging fine-mapping approaches should be combined with the one shown here to optimize the portability across populations.

## Conclusions

Given the low portability of PRS across populations, we investigated the potential of PTRS to help address the issue. We found that with the current transcriptome reference datasets restricted to cis regions for gene expression imputation, the phenotypic prediction performance of PTRS was lower than that of PRS. However, we also found that the prediction performance was higher than what could be expected given the much-reduced set of predictors (on the order of 10-20 thousand genes instead of several million SNPs) and the total variance explained by the predicted transcriptome. The portability, defined as the performance relative to the disease risk prediction in the European-descent target set, was higher for PTRS than PRS in the African-descent target set, where the loss of PRS performance is most pronounced. We hypothesize that these results reflect the conserved role of genes in disease even when the SNPs that predict disease differ between ancestry groups and suggest that integrating PTRS can be a useful tool to improve the transfer of PRS across populations. Corroborating this idea, we found that the combined PRS and PTRS had higher performance and portability in the African ancestry target set. The effect was not significant in other ancestry groups highlighting the need to continue improving the transcriptome prediction models as well as PTRS training.

A critical advantage of PTRS is the clear biological interpretation—it provides information about how genes are related to a trait. Unlike PRS, PTRS is grounded in biology and can serve as a building block for future extensions. Also, tantalizing is the possibility of using PTRS to port polygenic signals not just over ancestry- but also species-boundaries. Finally, our results suggest that collecting a more diverse transcriptomic and other omic reference data could be a cost effective way to improve portability across populations.

## Methods

### Obtaining individuals and phenotypes from UK Biobank

We used data from UK Biobank downloaded on July 19, 2017. We excluded related individuals and the ones with high missing rate or other sequencing quality issues. As covariates, we extracted age at recruitment (Data-Field 21022), sex (Data-Field 31), and the first 20 genetic PCs. The ancestry information of individuals was obtained from Data-Field 21000, and we kept individuals labeled as “British,” “Indian,” “Chinese,” or “African” (according to Data-Coding 1001: http://biobank.ctsu.ox.ac.uk/crystal/coding.cgi?id=1001). Throughout the paper, we labeled ‘British’ individuals as EUR, “Indian,” “Bangladeshi,” and “Pakistani” individuals as S.ASN, “Chinese” individuals as E.ASN, and “African” and “Caribbean” individuals as AFR. The measurements of the 17 quantitative phenotypes (as shown in Additional file 1: Table S1) across all available instances and arrays were retrieved. The data retrieval described above was performed using ukbREST [21] with the query YAML file available at https://github.com/liangyy/ptrs-ukb/blob/master/output/query_phenotypes.yaml[22].

If one individual has multiple measurements for the same phenotype (in more than one instances and/or more than one arrays), we collapsed multiple arrays by taking the average and we aggregated measurements across multiple instances by taking the first non-missing value. Individuals with missing phenotype in any of the 17 quantitative phenotypes or covariates were excluded.

### Quality control on self-reported ancestry

To ensure the quality of ancestry label, we removed individuals who deviate substantially from the population that they were assigned to. Specifically, for population *k* among the 4 populations (EUR, S.ASN, E.ASN, and AFR), we treated the distribution of the individuals, in the space of the first 10 PCs, as multivariate normal. And we calculated the observed population mean $\widehat {\mu }_{k}$ and covariance $\widehat \Sigma _{k}$ accordingly. Then, for each individual *i* in population *k*, we evaluated the “similarity” *S*_*ik*_ to the population *k* as $S_{ik} = \log \Pr (\text {PC}_{i}^{1}, \cdots, \text {PC}_{i}^{10}; \widehat {\mu }_{k}, \widehat {\Sigma }_{k})$. Intuitively, if an individual has genetic background differing from is the assigned population, the corresponding *S*_*ik*_ will be much larger than others. So, we filtered out individuals with *S*_*ik*_≤−50 in the assigned population *k*. This cutoff was picked such that $\phantom {\dot {i}\!}S_{ik^{\prime }}$ for any un-assigned population *k*^′^ has $S_{ik^{\prime }} \le -50$ for all individuals.

The number of individuals remained after data retrieval and ancestry quality control is listed Additional file 1: Table S2.

### Performing GWAS and building LD clumping and *p*-value thresholding based PRS models

We built PRS using the genotypes and phenotypes of the individuals in the discovery data set (the details of data splitting is described in the “Experimental setup” section). We performed GWAS (linear regression) using linear_regression_rows in hail v0.2 where we included covariates: first 20 genetic PCs, age, sex, age^2^,sex×age, and sex×age^2^. In the GWAS run, we excluded variants with minor allele frequency < 0.001 and variants that significantly deviate from Hardy-Weinberg equilibrium (*p*-value < 10^−10^). And the phenotype in their original scales were used.

To obtain relatively independent associations for PRS construction, we ran LD clumping using plink1.9 with option –clump –clump-p1 1 –clump-r2 0.1 –clump-kb 250. This command extracted genetic variants in the order of their GWAS significances and excluded all variants having *R*^2^>0.1 to or 250 kb within any variants that have already been included. The PRS was constructed on the basis of the set of variants obtained from the LD clumping along with the marginal effect size estimated in GWAS run. Specifically, we calculated PRSs at a series of GWAS *p*-value thresholds: 5×10^−8^,10^−7^,10^−6^,10^−5^,10^−4^,10^−3^, 0.01, 0.05, 0.1, 0.5, and 1. In other words, at threshold *t*, the PRS for individual *i* was calculated as

1 $$\begin{array}{*{20}l} \text{PRS}_{i}^{t} &= \sum_{j: p_{j} \le t} X_{ij} \widehat{b}_{j},  \end{array} $$

where *X*_*ij*_ is the effect allele dosage of variant *j* in individual *i* and $\widehat {b}_{j}$ is the estimated effect size of variant *j* from GWAS run.

At the testing stage, given the genotype of an individual, we calculated the PRS of the individual using Eq. 1.

### Computing the predicted transcriptome

We computed predicted gene expression for all individuals passing filtering steps and quality control. We utilized two sets of prediction models: (1) CTIMP models (proposed in [11]) trained on GTEx v8 EUR individuals [23] and (2) elastic net models which were trained on Europeans (EUR) or African Americans in combination with Hispanics (AFHI) [15]. The sample size and tissue information of the prediction models are listed in Additional file 1: Table S3.

### Estimating PVE by predicted transcriptome of a single tissue

To assess the potential predictive power of predicted transcriptome on the phenotypes of interest, we estimated the proportion of phenotypic variation that could be explained by the predicted transcriptome in aggregate. Specifically, we assume the following mixed effect model (for individual *i*).

2 $$\begin{array}{*{20}l} Y_{i} &= \mu + \sum_{l} C_{il} a_{l} + \sum_{g} \widetilde{T}_{ig} \beta_{g} + \epsilon_{i}  \end{array} $$

3 $$\begin{array}{*{20}l} \epsilon_{i} &\sim_{iid} N(0, \sigma_{e}^{2}) \end{array} $$

4 $$\begin{array}{*{20}l} \beta_{g} &\sim_{iid} N(0, \frac{\sigma_{g}^{2}}{M}), \end{array} $$

where *M* denotes the number of genes, *C*_*il*_ is the *l*th covariate, $\widetilde {T}_{ig}$ is the inverse normalized predicted expression for gene *g*, and *Y*_*i*_ is the observed phenotype. By inverse normalization, we converted the predicted expression $\widehat {T}_{ig}$ to $\widetilde {T}_{ig}$ by $\widetilde {T}_{ig} = \Phi ^{-1}(\frac {\text {rank}(\widehat {T}_{ig})}{N + 1})$ within each gene *g* where *N* is the number of individuals and ‘rank’ is in increasing order, so that we have $\widetilde {T}_{ig} \sim N(0, 1)$. The parameters of the model were estimated using hail v0.2 stats.LinearMixedModel.from_kinship with *K* matrix being set as $\widetilde {T} \widetilde {T}^{t} / M$. And PVE is calculated as $\frac {\hat \sigma _{g}^{2}}{\hat \sigma _{e}^{2} + \hat \sigma _{g}^{2}}$. The same set of covariates as the ones for PRS were used.

The PVE estimation was performed for each transcriptome model and population pairs. For non-European populations, all individuals were included in the analysis. We randomly selected 5,000 EUR individuals for the analysis.

### Estimating PVE by predicted transcriptome of multiple tissues

The genetic effects on the complex trait can be mediated through the regulation of expression in different tissues so that including predicted transcriptomes in multiple tissues could potentially improve the prediction performance. To quantify the gain, we performed the PVE analysis as described in the previous section using predicted expression in 10 GTEx tissues (listed in Additional file 1: Table S3) instead of just one. To avoid colinearity issues caused by the high correlation of predicted expression among tissues, we used eigenvectors of the predicted transcriptome matrix instead of the actual predicted expression. More specifically, for each gene *g*, we formed the matrix of predicted expression across the 10 tissues and performed singular value decomposition. We only retained eigenvectors with singular values that were at least 1/30 of the maximum singular value of the gene’s expression matrix across tissue. This approach is similar to the one used for combining PrediXcan association in multiple tissues [24].

### Estimating chip heritability

The chip heritability was estimated using the mixed effect model which is similar to the one for PTRS PVE estimation. But here we replace predicted transcriptome with genome-wide variant genotypes. The same cohorts and covariates were used as the ones in PTRS PVE estimation.

### Transcriptome prediction models for PTRS construction

The predicted transcriptome in the discovery set (UKB EUR) was calculated using models from GTEx [23] and MESA EUR based models [15] listed in Additional file 1: Table S3). The GTEx EUR whole blood transcriptome consisted of 7,041 genes. For the MESA transcriptomes, we restricted the prediction to the 4041 genes that were present in both the MESA EUR models and the MESA AFHI models (to ensure that PTRS built in the discovery set with the EUR models could be computed without missing genes in the target sets using the AFHI models).

### Building PTRS models using elastic net

For each of the 17 quantitative phenotypes, we trained elastic net model to predict the phenotype of interest using the predicted transcriptome (in a single tissue) as features. The same set of covariates as the ones for PRS were used. Let $\widehat {T}_{g} \in \mathbb {R}^{N \times 1}$ denote the standardized predicted expression level of gene *g* across *N* individuals. Similarly, let $C_{l} \in \mathbb {R}^{N \times 1}$ denote the observed value of the *l*th standardized covariate. We fit the following elastic net model.

5 $$\begin{array}{*{20}l} \beta^{\text{EN}} &= \arg\min_{\beta} \overbrace{\frac{1}{N} \| Y - X \beta - \beta_{0} \|_{2}^{2}}^{\text{loss:} l(\beta)} + \lambda \alpha \|\beta\|_{1} + \lambda (1 - \alpha) \|\beta\|_{2}^{2}  \end{array} $$

6 $$\begin{array}{*{20}l} X &:= [ \widehat{T}_{1}, \cdots, \widehat{T}_{M}, C_{1}, \cdots, C_{L} ] ~, \end{array} $$

where *β*_0_ is the intercept, *M* is the number of genes, *L* is the number of covariates, $\|\beta \|_{2}^{2}$ is the *l*_2_ norm, and ∥*β*∥_1_ is the *l*_1_ norm of the effect size vector. Here, *α* controls the relative contribution of the *l*_1_ penalization term and *λ* controls the overall strength of regularization.

The model fitting procedure was implemented using tensorflow v2 with mini-batch proximal gradient method and the code is available at https://github.com/liangyy/ptrs-tf [25]. We trained models at *α*=0.1 (*α* = 0.5 and 0.9 show similar performance). And fixing the *α* value, as suggested in [26], we trained a series of models for a sequence of *λ*’s starting from the highest. The maximum *λ* value, *λ*_max_, was determined as the smallest *λ* such that Eq. 7 is satisfied.

7 $$\begin{array}{*{20}l} |~ \nabla l(\beta) ~| \le \alpha \lambda,  \end{array} $$

where the gradient is evaluated at

8 $$\begin{array}{*{20}l} \beta_{0} = \overline{Y}, \beta_{\text{covariate}} = \mathbf{0}, \beta_{\text{transcriptome}} = \mathbf{0}  \end{array} $$

So, at *λ*=*λ*_max_, Eq. 8 is the solution to Eq. 5, which could serve as the initial points for the subsequent fittings of *λ*’s. We estimated *λ*_max_ using the first 1000 individuals of the data. And the sequence of *λ* was set to be 20 equally spaced points in log scale with the maximum value being 1.5*λ*_max_ and the minimum value being *λ*_max_/10^4^. Among these PTRS models generated at different *λ* values, we only kept the first 11 non-degenerate PTRS models so that we have the same number of candidate models for both PRS and PTRS.

### Building PTRS models using the LD clumping and *p*-value thresholding based approach

To implement clumping and thresholding based PTRS, we first obtained the gene/phenotype associations (PrediXcan association [9]) by associating the predicted gene expression to the phenotypes with the same set of covariates as PRS models. Next, we performed gene-based LD clumping to extract the roughly independent genes for PTRS models. The LD clumping procedure is described as follows. First, we prioritized genes according to their PrediXcan *p*-values. Second, we traversed the prioritized gene list from the most significant ones to the least ones, in which we included a gene into the returned gene list if the squared correlation (in terms of the predicted expression) of this gene and any other genes that have been included in the current returned gene list is smaller than 0.1.

Among these genes being selected in LD clumping, we built PTRS models by *p*-value thresholding. Specifically, at *p*-value thresholds 10^−6^,5×10^−6^,10^−5^,5×10^−5^,10^−4^,5×10^−4^,10^−3^,5×10^−3^, 0.01, 0.05, 0.1, 0.5, 1, we built a PTRS model by keeping the genes with *p*-values smaller than the cutoff. For these genes being included in the final PTRS model, we used the effect sizes estimated in the PrediXcan association as the PTRS weights.

### Calculating PTRS in target sets

At the testing stage, given the standardized (within the population) predicted transcriptome of an individual, we calculated the PTRS of the individual using the following:

9 $$\begin{array}{*{20}l} \text{PTRS}_{i}^{\lambda} &= \sum_{g} \widehat{T}_{ig} \beta_{g}^{\lambda},  \end{array} $$

where *β*^*λ*^ denotes the *β*^EN^ obtained at hyperparamter equal to *λ*. For the PTRS built upon from GTEx EUR predicted transcriptome, the target PTRS was calculated with the GTEx EUR transcriptome (transcriptome predicted with GTEx EUR gene expression prediction models). To examine the utility of population-matched prediction model, the PTRS on the target set were calculated with of both MESA EUR and MESA AFHI transcriptomes.

### Quantifying the prediction accuracy of PRS and PTRS with partial *R*^2^ ($\widetilde {R}^{2}$)

To measure the predictive performance of PRS and PTRS, we calculated the partial *R*^2^ of PRS/PTRS against the observed phenotype accounting for the set of covariates listed in Methods. Specifically, for individual *i*, let $\hat {y}_{i}$ denote the predicted phenotype which could be either PRS or PTRS and *y*_*i*_ denote the observed phenotype. Partial *R*^2^ (denoted as $\widetilde {R}^{2}$ below) is defined as the relative difference in sum of squared error (SSE) between two linear models: 1) *y*∼1+covariates (null model); and 2) $y \sim 1 + \text {covariates} + \hat {y}$ (full model), *i.e.*$\widetilde {R}^{2} = 1 - \frac {\text {SSE}_{\text {full}}}{\text {SSE}_{\text {null}}}$. To enable fast computation, we calculated $\widetilde {R}^{2}$ using an equivalent formula shown in Eq. 10 which relies on the projection matrix of the null model.

10 $$\begin{array}{*{20}l} \widetilde{R}^{2} &= \frac{\mathcal{C}^{2}(y, \hat{y})}{\mathcal{C}(y, y)\mathcal{C}(\hat{y}, \hat{y})}  \end{array} $$

11 $$\begin{array}{*{20}l} \mathcal{C}(u, v) &:= u^{t} v - u^{t} H v, \end{array} $$

where *H* is the projection matrix of the null model, *i.e.*$H = \widetilde {C} (\widetilde {C}^{t} \widetilde {C})^{-1} \widetilde {C}^{t}$ where $\widetilde {C} = [1, C_{1}, \cdots, C_{L}]$ with *C*_*l*_ being the *l*th covariate.

As stated in the results section, PTRS weights were computed in the discovery set (UKB EUR) and tested in the 5 target sets. To determine the hyperparameter (*p*-value cutoff in the clumping and thresholding based approach or *λ* value in elastic net), for each target set, we further split the target set into two equal-size parts, a validation set and a test set. First, we calculated $\widetilde {R}^{2}$ for all hyperparameters in the validation set and we selected the hyperparameter that maximized the $\widetilde {R}^{2}$ in the validation set. And then, we calculated the $\widetilde {R}^{2}$ under the selected hyperparameter in the test set. This procedure was repeated 10 times and we reported the average $\widetilde {R}^{2}$ in the test set as the prediction accuracy.

### Combining PTRS and PRS

We combined PTRS and PRS as a weighted sum, i.e., combined score=*c*_1_PRS+*c*_2_PTRS with *c*_1_ and *c*_2_ calibrated in a validation subset of the target set. Given a sequence of PTRSs and PRSs at different hyperparameters (trained with the discovery set, UKB EUR), we used the following procedure to obtain the prediction accuracy in each of the ancestry groups. Similar to the prediction accuracy quantification of PTRS and PRS alone, we first split the target set into validation and test sets. We used the validation set to the following: (i) determine the coefficients (*c*_1_ and *c*_2_) for each PTRS’ and PRS’ hyperparameter combination and (ii) select the best combined score among all PTRS and PRS combinations. And the test set was used to obtain an unbiased measure of the prediction accuracy of the selected score in the corresponding ancestry. To achieve the two goals above using the validation set, we further split the validation set into two equal-size parts. Using the first half of the validation set, we determined the coefficients (*c*_1_ and *c*_2_) for each of the PTRS and PRS combinations by minimizing the square difference between predicted and observed. Next, using the other half of the validation set, we calculated the prediction accuracy of each PTRS and PRS combination with the coefficients obtained above. We note that this accuracy measure is unbiased since neither PTRS and PRS weights nor the coefficients for combining PTRS and PRS were obtained using this data. Based on these prediction accuracy measures, for each ancestry, we selected the best-performing PRS and PTRS combination along with the corresponding coefficients. We report the prediction performance calculated in the test set using the optimal combined score selected in the validation set.

### Quantifying the portability of PRS and PTRS

Portability was defined as the ratio of the prediction accuracy in each target set divided by the prediction accuracy in the European reference set. Therefore, by definition, portability in the EUR ref. set was 1.

When calculating the portability of PTRS using MESA AFHI transcriptome, we used the MESA EUR model $\widetilde {R}^{2}_{\text {EUR ref.}}$ as the reference. This is a conservative choice since MESA EUR model is expected to perform better than MESA AFHI model among EUR individuals.

## Code and data availability

Code for data analysis is on Zenodo with the access code DOI: (10.5281/zenodo.5709387) [27] and GitHub (https://github.com/liangyy/ptrs-ukb) [22]. Code for mini-batch elastic net is on Zenodo with the access code DOI: (10.5281/zenodo.5709389) [28] and GitHub (https://github.com/liangyy/ptrs-tf) [25]. Prediction models for gene expressions from GTEx v8 and MESA study are available at https://predictdb.org [29]. The chip heritability and PVE analysis results are at Additional file 2: Table S4 and Additional file 3: Table S5. And the PRS and PTRS $\widetilde {R}^{2}$ results are at Additional file 4: Table S6 and Additional file 5: Table S7.

## Supplementary Information

**Additional file 1** Tables S1-S3, legends for Tables S4-S7, and Figures S1-S7.

**Additional file 2** Table S4.

**Additional file 3** Table S5.

**Additional file 4** Table S6.

**Additional file 5** Table S7.

**Additional file 6** Review history.
