# Supplementary material for: Polygenic transcriptome risk scores (PTRS) can improve portability of polygenic risk scores across ancestries
Source: Genome Biol. 2022 Jan 13;23:23. doi: 10.1186/s13059-021-02591-w (PMC8759285; doi:10.1186/s13059-021-02591-w)
Supplement: Supplementary file 1 — Additional file 1 Tables S1-S3, legends for Tables S4-S7, and Figures S1-S7. [file 13059_2021_2591_MOESM1_ESM.pdf]

Supplementary Tables and Figures for
Polygenic transcriptome risk scores (PTRS) can improve portability of polygenic risk scores across ancestries

Yanyu Liang   Milton Pividori   Ani Manichaikul   Abraham A. Palmer
Nancy J. Cox   Heather Wheeler   Hae Kyung Im

November 23, 2021

1 Supplementary tables

UKB Field Description	UKB Field ID	Tag	Phenotype Category
Standing height	50	Height	Height
Diastolic blood pressure, automated reading	4079	DBP	Blood pressures
Systolic blood pressure, automated reading	4080	SBP	Blood pressures
Body mass index (BMI)	21001	BMI	BMI
White blood cell (leukocyte) count	30000	WBC	Blood cell counts
Red blood cell (erythrocyte) count	30010	RBC	Blood cell counts
Haemoglobin concentration	30020	Hb	Haemoglobin related
Haematocrit percentage	30030	Ht	Haemoglobin related
Mean corpuscular volume	30040	MCV	Haemoglobin related
Mean corpuscular haemoglobin	30050	MCH	Haemoglobin related
Mean corpuscular haemoglobin concentration	30060	MCHC	Haemoglobin related
Platelet count	30080	Platelet	Blood cell counts
Lymphocyte count	30120	Lymphocyte	Blood cell counts
Monocyte count	30130	Monocyte	Blood cell counts
Neutrophill count	30140	Neutrophil	Blood cell counts
Eosinophill count	30150	Eosinophil	Blood cell counts
Basophill count	30160	Basophil	Blood cell counts

Table S1: **Meta information of the phenotypes retrieved from UK Biobank which were used in the analysis**. The “Tag” column shows the short name of the phenotyes used in this paper. And phenotypes are assigned into five categories which are shown in “Phenotype Category” column

Ancestry	Number of individuals
AFR	6413
EUR	356476
E.ASN	1326
S.ASN	6479

Table S2: **Number of individuals included in the analysis stratified by ancestry**.

Method	Data source	Population	Tissue	Number of genes	Sample size	Tag
CTIMP	GTEx V8	European	Adipose_Subcutaneous	9228	491	
CTIMP	GTEx V8	European	Artery_Tibial	9027	489	
CTIMP	GTEx V8	European	Breast_Mammary_Tissue	8127	337	
CTIMP	GTEx V8	European	Cells_Cultured_fibroblasts	8731	417	
CTIMP	GTEx V8	European	Lung	8954	444	
CTIMP	GTEx V8	European	Muscle_Skeletal	7671	602	
CTIMP	GTEx V8	European	Nerve_Tibial	10184	449	
CTIMP	GTEx V8	European	Skin_Sun_Exposed_Lower_leg	9474	517	
CTIMP	GTEx V8	European	Thyroid	9827	494	
CTIMP	GTEx V8	European	Whole_Blood	7041	573	GTEx EUR
Elastic Net	MESA		Monocyte	4670	578	MESA EUR
Elastic Net	MESA	African American or Hispanic	Monocyte	5554	585	MESA AFHI

Table S3: **Meta information of the prediction models used in the analysis**. The highlighted prediction models were used to build PTRS. The "Tag" column shows the short name of the models used in this paper.

2 Supplementary tables as additional files

The tables are attached as "Additional files" of the paper. The legends of these tables are outlined here.

Table S4: **Chip heritability of the 17 quantitative traits in UK Biobank via REML.** (See Additional file 2) Column "chip_h2" shows the observed h2 in REML. Column "chip_h2_se" shows the standard error of chip_h2.

Table S5: **The proportion of phenotypic variation explained (PVE) by the predicted transcriptome of the 17 quantitative traits in UK Biobank.** (See Additional file 3) Column "num_genes" shows the number of genes (or independent predictors for multi-tissue case) in the transcriptome model. Columns "pve" and "pve_se" show the PVE estimate and corresponding standard error based on linear mixed effect model. Column "population" shows the target population. Column "train_population" shows the population that the transcriptome model is trained on. Column "training_data" shows the training data source of the transcriptome models. Column "tissue" shows the tissue type of the trainscriptome. (the "10 Tissues" is listed in Table S3).

Table S6: **Prediction performance of PRS and PTRS based on GTEx whole blood models.** (See Additional file 4) For each target population the partial R2 of the PRS and PTRS is shown. Column "PRS" shows the partial R2 of clumping and thresholding based PRS. Column "(EN) PTRS" shows the partial R2 of elastic net based PTRS. Column "(EN) PTRS+PRS" shows the partial R2 of the combined score based on PRS and elastic net based PTRS. Column "(CT) PTRS" shows the partial R2 of clumping and thresholding based PTRS. Column "(CT) PTRS+PRS" shows the partial R2 of the combined score based on PRS and clumping and thresholding based PTRS.

Table S7: **Prediction performance of PTRS based on MESA monocyte models.** (See Additional file 5) For each testing population (target population) the partial R2 of the PTRS is shown. PTRS is calculated as PTRS = $\sum_{g \in \text{genes}} W_g \times E_g$ with W_g being the weight of gene g and E_g being the predicted expression of gene g. Column "PTRS (MESA EUR)" shows the partial R2 of the PTRS with W_g trained with MESA EUR models and E_g is also based on MESA EUR models. Column "PTRS (MESA AFHI)" shows the partial R2 of the PTRS with W_g trained with MESA EUR models (limiting to genes that also occur in MESA AFHI models) and E_g is also based on MESA AFHI models. Column "PTRS (MESA ALL)" shows the partial R2 of the PTRS with W_g trained with MESA ALL models and E_g is also based on MESA ALL models.

3 Supplementary figures

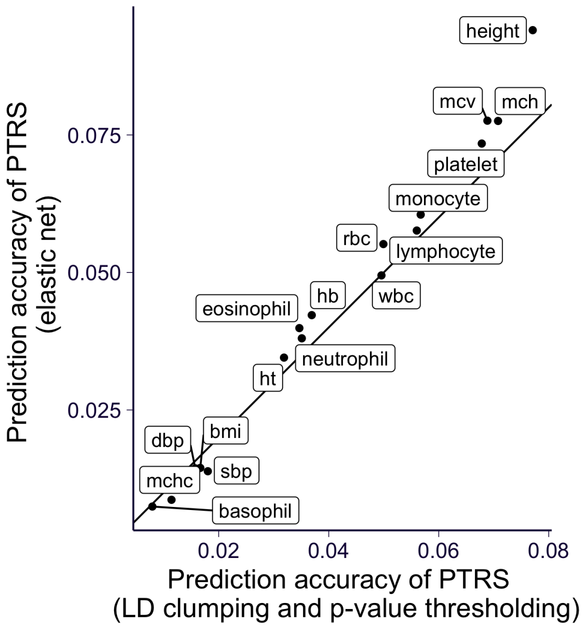

Fig. S1: **Prediction accuracy of PTRS built with the elastic nets vs the LD clumping and p-value thresholding approach.** The prediction accuracy of PTRS built with the LD clumping and p-value thresholding approach was shown on x-axis. And the accuracy of PTRS built with the elastic net was shown on y-axis. The PTRS construction was based on the transcriptome models from GTEx EUR whole blood samples.

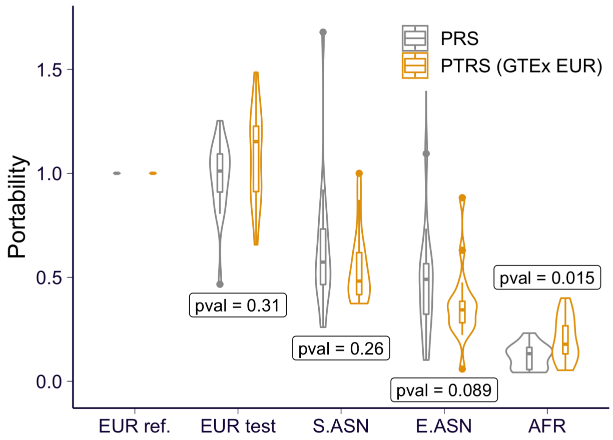

Fig. S2: **Portability of LD clumping and p-value thresholding based PTRS for 17 quantitative phenotypes in UK Biobank.** The portability of clumping and thresholding based PTRS trained and calculated using GTEx EUR whole blood samples are shown in yellow with the PRS shown in gray. 'EUR ref.' set is used as the reference population in the calculation of portability so that the portability is always 1.

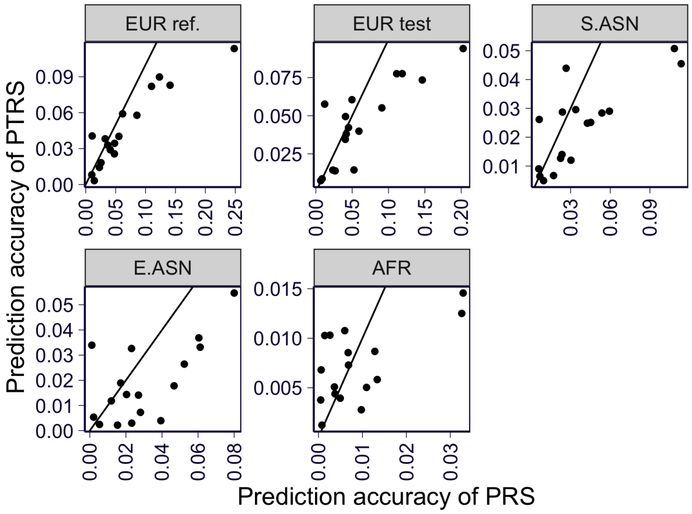

Fig. S3: **Prediction accuracy of PTRS vs PRS in all ancestral groups.** Prediction accuracy, measured by partial $\widetilde{R}^2$, of PTRS (on y-axis) was compared to the accuracy of PRS (on x-axis). Each panel corresponds to each target set. The PTRS construction was based on the transcriptome models from GTEx EUR whole blood samples.

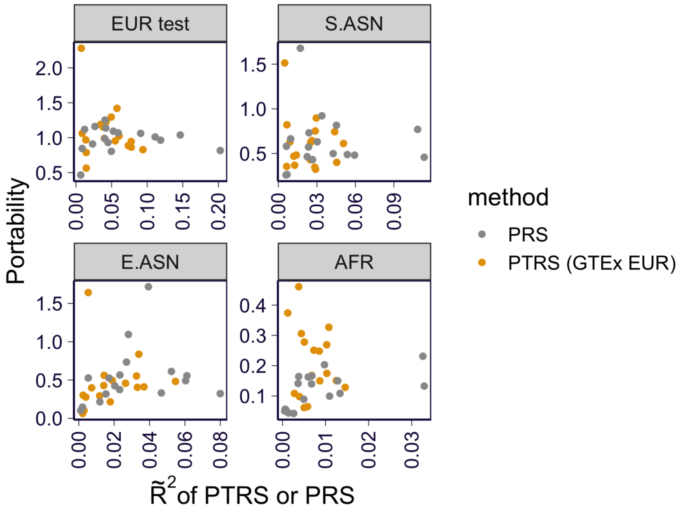

Fig. S4: **Prediction accuracy vs portability of PTRS in all ancestral groups.** Portability of PTRS (y-axis) was compared to the prediction accuracy, measured by partial $\widetilde{R}^2$, of PTRS (on x-axis). Each panel corresponds to each target set. The PTRS construction was based on the transcriptome models from GTEx EUR whole blood samples.

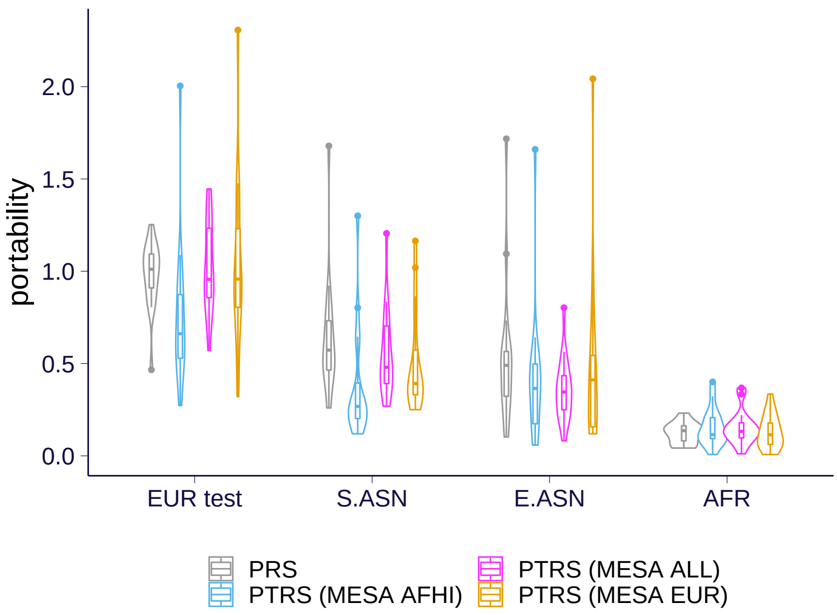

(a)

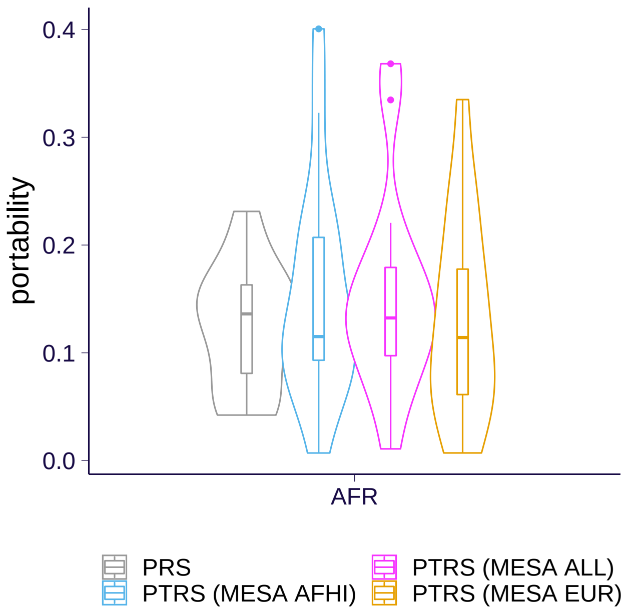

(b)

Fig. S5: **Portability of PRS and MESA-based PTRSs.** The results of the 17 quantitative traits are summarized in the violin and box plots for each of the score types. **(A)** Results in all ancestry groups are shown. **(B)** A zoom-in plot focusing on results in African ancestry.

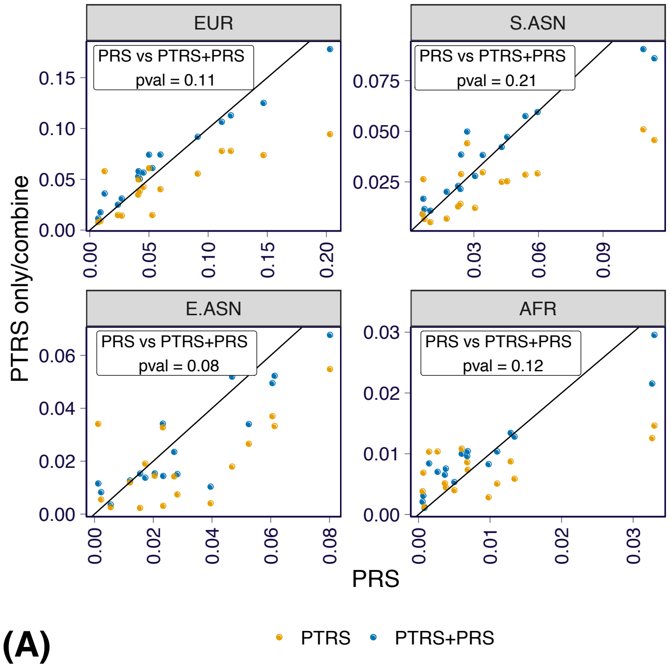

(A)

(B)

sample	R2 (PRS – PTRS)	wilcox_pval
AFR	−0.000937	0.1200
EUR test	−0.004660	0.1090
EUR ref.	−0.000912	0.3060
E.ASN	0.004920	0.0797
S.ASN	−0.001040	0.2070

Fig. S6: **Prediction accuracy of the score combining PTRS and PRS.** Combing the clumping and thresholding-based PTRS and PRS, the results on the prediction accuracy are shown below. **(A)** The prediction accuracy of the PRS is shown on x-axis and it is compared against the prediction accuracy of the PTRS (yellow) or the combined score on y-axis. The results on all of the 17 quantitative traits are shown. Each panel corresponds to one ancestry group. The p-values are for comparing PRS accuracy versus the combined score accuracy via the paired Wilcoxon signed rank test. **(B)** A summary of the difference between the prediction accuracy of PRS and the combined score is shown for each of the ancestry group. The second column shows the mean difference and the third column shows the results of the paired Wilcoxon signed rank test comparing the accuracy of PRS versus the combined score.

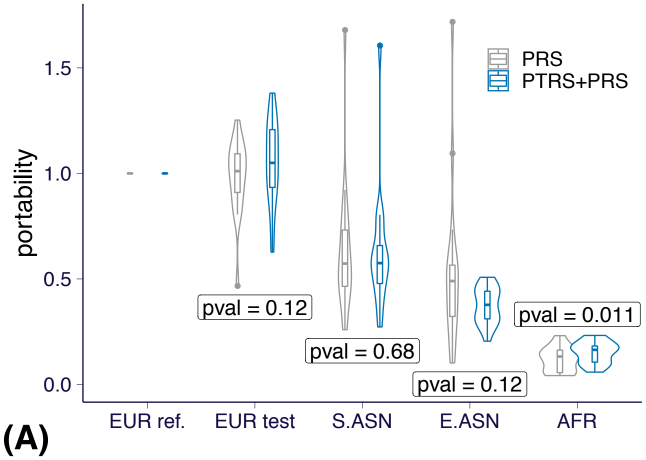

(A)

sample	portability (PRS – PTRS)	wilcox_pval
AFR	–0.03280	0.011
EUR test	–0.07350	0.120
E.ASN	0.15900	0.120
S.ASN	0.00408	0.678

(B)

Fig. S7: **Portability of the score combining PTRS and PRS.** Combing the clumping and thresholding-based PTRS and PRS, the results on the portability are shown below. **(A)** The prediction accuracy of the PRS is shown on x-axis and it is compared against the prediction accuracy of the PTRS (yellow) or the combined score on y-axis. The results on all of the 17 quantitative traits are shown. Each panel corresponds to one ancestry group. The p-values are for comparing PRS accuracy versus the combined score accuracy via the paired Wilcoxon signed rank test. **(B)** A summary of the difference between the prediction accuracy of PRS and the combined score is shown for each of the ancestry group. The second column shows the mean difference and the third column shows the results of the paired Wilcoxon signed rank test comparing the accuracy of PRS versus the combined score.
