# Supplementary material for: Polygenic transcriptome risk scores (PTRS) can improve portability of polygenic risk scores across ancestries
Source: Genome Biol. 2022 Jan 13;23:23. doi: 10.1186/s13059-021-02591-w (PMC8759285; doi:10.1186/s13059-021-02591-w)
Supplement: Supplementary file 6 — Additional file 6 Review history. [file 13059_2021_2591_MOESM6_ESM.docx]

Review History

**First round of review**

**Reviewer 1**

**Were you able to assess all statistics in the manuscript, including the appropriateness of statistical tests used?**

**Yes, and I have assessed the statistics in my report.**

**Comments to author:**

- Are the methods appropriate to the aims of the study, are they well described, and are necessary controls included? If not, please specify what is required.

The proposed PTRS is not the preferred approach for resolving the issue of translating GWAS results or phenotype risk prediction from one ethnicity group to another.

- Are the conclusions adequately supported by the data shown? If not, please explain

No. The PTRS is not shown to outperform the PRS approach for European and Asian groups. And the recently proposed PRS methods should be considered in this comparison.

- Are sufficient details provided to allow replication and comparison with related analyses that may have been performed? If not, please specify what is required.

Yes.

- Does the work represent a significant advance over previously published studies?

No. See my comments below.

- Is the paper of broad interest to others in the field, or of outstanding interest to a broad audience of biologists?

Yes for the topic of translating GWAS findings across ethnicity groups. No for the proposed approach.

Comments:

Even though it is interesting for this paper to derive a polygenic transcriptome risk score (PTRS) to investigate the predictivity of genetically regulated gene expression values for phenotypes and how this PTRS might be portability across different ethnicity groups, I think the recently proposed PRS methods including the PRS-CS method (G. Tian et. al, nature Communications, 2019, https://www.nature.com/articles/s41467-019-09718-5) accounting for LD and XP-BLUP (Coram et al. AJHG 2017, 10.1016/j.ajhg.2017.09.005 ) modeling discovery and test GWAS summary statistics data sets that are of different ethnicities are preferred ways to translate GWAS summary statistics obtained in a discovery data set to another test GWAS summary statistics data set of different ethnicity by deriving polygenic risk scores. Here are my main concerns:

1. Especially, given the subtle difference between the phenotype risk predictivity by PTRS and the standard PRS with respect to "portability" as shown in the paper, I think it is worth to compare with the PRS derived by PRS-CS with tunning parameters determined by the test dataset and the XP-BLUE method.

2. As shown from the trained gene expression prediction models by PrediXcan with the GTEx reference data, only ~5000 genes would have a valid prediction model per tissue type. The poor performance of accounting <50% SNP heritability by PTRS and not outperforming PRS in test data of European and Asian ethnicities might due to not accounting for causal SNPs whose effects on phenotype are not mediated through transcriptome. This also show the proposed PTRS should not be used alone for predicting phenotype risks.

3. Since the SNP heritability is known not uniformly distributed over all genome-wide SNPs as shown by all GWAS results, comparing PVE due to predicted gene expression to the "expected" phenotype heritability does not make sense. The P+T standard PRS method might even show a higher fold of difference with respect to the "expected" phenotype heritability.

4. As mentioned by the authors, using the same test data to tune prediction model for both P+T PRS or PTRS is not suitable in practice. One could use half of the test GWAS data as a validation data set to tune these parameters, and then test on the other half of the test GWAS data.

**Reviewer 2**

**Were you able to assess all statistics in the manuscript, including the appropriateness of statistical tests used?**

**Yes, and I have assessed the statistics in my report.**

**Comments to author:**

This paper by Liang et al. proposes a new method to leverage genetically predicted gene expression levels by integrating them into a polygenic transcriptome risk score (PTRS) that can be used for complex trait prediction. The authors show that the PTRS have slightly weaker predictive ability compared to normal PRS trained using GWAS weights. However, the authors hypothesized the predicted transcriptome information may be more consistent across populations than GWAS-weights and test whether PTRS trained using European transcriptome data may have improved prediction in other ancestries compared to PRS. The authors show that these PTRS are at least as portable as PRS across ancestry groups while being slightly more portable than PRS in African ancestry participants (whose genetic distance is highest from the training population). Overall I think that this is an illustrative study about how gene-expression information may improve portability; however, I outline some concerns that I think can easily be addressed with additional analyses of the data at hand.

Major comments:

-My primary concern with the paper's methodology is that the PRS and PTRS are optimized for each target population (lines 169-71), leaving each target set to have a different set of genes or variants in the model. This is a problem for two reasons: (1) it is dissimilar from traditional PRS analyses the p-value threshold is exclusively learned in the discovery or tuning dataset - the performance (e.g. r2) should only be measured for external validation samples to avoid inflated performance metrics, (2) PRS transferability is usually compared using the single model (e.g. single p-value/LD-threshold) that is applied and evaluated in multiple populations. My interpretation of the current analysis is that it measured the portability of the underlying predictdb data rather than actual scores since this paper uses a target-sample specific p-value threshold (PRS) or lambda value (PTRS) - this is also inconsistent with the terms 'weights from UK Biobank Discovery Set' labels in Figure 1. I think the analysis needs to be redone with a single PRS and PTRS learned in the discovery and EUR ref datasets that are then evaluated as-is in the different ancestry groups to make the specific claims about score portability.

-The current display of the data in Figure 4A may be misleading to some readers, as it shows the predictive accuracy of the EUR ref PTRS as equivalent to the EUR ref PRS. The figure legend and text should be clarified to reflect this, as it doesn't accurately display that the PTRS underperforms the PRS across ancestries. Perhaps including a version of Figure 3A comparing PRS vs. PTRS using the GTex EUR data in all target datasets might adequately fulfill this criterion, such that you see the absolute comparison of PRS/PTRS in each ancestry as well as the relative performances in the current figure 4a. In addition, I think Figure 4B should be extended to all target ancestries so that readers can see if the GTex result is consistent across all ancestries using MESA EUR data as the improved portability claim is only made using p-values of t-tests in the African target sample (tail of the genetic distance distribution) rather than using some sort of trend-test of the decline across strata.

-What is the rationale behind not using the MESA ALL ancestry data as a source of data? Multi-ancestry GWAS SumStats usually improve cross-population portability, it would be interesting to see if the same can be said of multi-ancestry genetically-predicted transcriptomes.

-The sample sizes of the non-European ancestry groups in UK Biobank are very small compared to previous analyses of the same data. Specifically, the number of African individuals is considerably smaller than seen in other papers (it seems you've excluded self-reported Caribbean identity), and the South Asian sample seems to have excluded British Pakastani and Bangladeshis? I'd suggest revisiting the inclusion criteria and rerunning the clustering to increase the sample size.

Minor comments:

-I found the data about which transcriptome is being predicted a little confusing throughout the manuscript. It is initially introduced as whole blood, but then Figure2A shows that including more Gtext tissues improves the PVE. But then the rest of the paper uses MESA and doesn't say the tissue. I wonder if the multi-tissue result should be presented as a final result/discussion point to motivate methodological developments and new data?

-How different are the genes selected in the PTRS across different ancestry groups and source datasets?

-It would be nice to have some mention of this paper's relationship to the related Gene Expression Risk Score method (Pain et al. 2021 doi:10.1093/hmg/ddab053).

-I wonder if the authors have access to disease phenotypes (CAD/Stroke/Diabetes)? How does the PTRS data perform at predicting binary phenotypes?

-Is portability the best word to describe performance differences across ancestries? Generalizability or transferability is more commonly seen in the polygenic score literature.

-The mention of cross-species trait prediction in the abstract seems unrelated to the current research. I suggest limiting this to the discussion, however, I think the use of PTRS in this scenario is still unrelated to transferability across populations.

**Reviewer 3**

**Were you able to assess all statistics in the manuscript, including the appropriateness of statistical tests used?**

**Yes, and I have assessed the statistics in my report.**

**Comments to author:**

This work proposes the development and use of a transcriptomic risk score (PTRS) model for trans-ethnic risk prediction. Rather than using SNPs to build a predictive scores (as in a PRS) the PTRS uses predicted expression to build a predictive score. The work investigates multiple aspects of the PTRS model. The PTRS features are shown to be substantially enriched for heritability. Perhaps the key result is that, although the PTRS has worse overall performance than the PRS, it carries over *relatively* better into non-European populations.

The manuscript is well written, tackles an interesting problem in a creative way, and is thorough. My main concerns are about the robustness of the primary result about PTRS portability. Given that the PTRS itself is not as accurate as the PRS, it is critical to show definitively that PTRS is more portable and understand why.

\# Major comments

* My main concern is the very modest improved portability of the PTRS vs PRS. In particular, the gain observed from GTEx EUR data into AFR (p=0.03) does not appear to replicate when using MESA EUR data (p=0.13) (Figure 4). Can the authors comment this lack of reproducibility? In Figure 4, some of the PTRS improvements appear to be outliers (i.e. a long tail of points with high y-values), are these outliers driving the significant difference? For example, does winsorizing the portability metrics retain the significant difference in portability? Given that portability is defined as a ratio, is it possible that traits with very low predictive accuracy are producing outsized portability ratios and adding more noise than signal? Is there anything interesting about individual traits that port over much better by PTRS than by PRS?

* The Discussion notes that the PTRS was trained using individual-level data and the PRS using summary-level data. It is also important to show that the improved portability is not simply a case of using a better training model for the PTRS. This could be addressed by retraining the PRS using individual-level data or retraining the PTRS using summary-level data to make this analysis consistent. Relatedly, the Results note that some in-sample parameter tuning was performed on the two scores (which is common), can the improved portability of the PTRS be an artifact of the in-sample tuning (i.e. the PTRS is a bit more overfit)? This could be addressed by performing a double cross-validation where the final target data is completely held out; or by using a prediction scheme that does not require in-sample parameter tuning.

* I'm puzzled by the proposed use case for this approach. Since one will always be able to generate a PRS if one has a PTRS, is there ever a case where improvement of the PTRS in isolation is meaningful? Isn't the metric of interest whether PRS+PTRS improves over PRS and/or improves in portability over PRS? Does PRS+PTRS do better in terms of portability than PRS alone? If PRS+PTRS is not better than PRS does this result have any practical application or is it more a biological/statistical curiosity?

\# Minor comments

* Figure 1 is very helpful!

* I'm puzzled by the use of LDSC regression to estimate SNP-heritability rather than using the exact variance components approach that was used to estimate T-heritability. From what I understand, LDSC regression is intended for estimating partitioned heritability and does not produce unbiased estimates of total heritability. Alternatively, it seems like the authors could have estimated the T-heritability using LDSC regression to make the apples-to-apples comparison.

* line 131: "This result is largely consistent to the estimates reported previously using a different approach" -- is this missing a figure/table?

* line 155: "For the European target set, the difference between using the MESA AFHI or the EUR transcriptomes was not significantly different from". This is interesting and surprising, can the authors comment on the asymmetry?

* line 175: These results seem consistent with T features capturing more heritability *per feature* than SNP features, and thus having less noise in the final score. There is a known relationship between heritability, GWAS sample size, number of SNPs, and expected prediction R^2 (for example: PMID 18852893). Can the authors comment on whether the R^2 they get from the PRS and PTRS are consistent with these expectations? This would help guide extrapolation to other PTRS studies.

**Authors Response**

**Point-by-point responses to the reviewers’ comments:**

Reviewer 1:

- Are the methods appropriate to the aims of the study, are they well described, and are necessary controls included? If not, please specify what is required.

The proposed PTRS is not the preferred approach for resolving the issue of translating GWAS results or phenotype risk prediction from one ethnicity group to another.

*Reply: We agree with the reviewer that we are not proposing a method that resolves the issue of translating GWAS results in general. The scope of the current manuscript is to provide proof of concept that integrating transcriptome regulation should be part of the strategy to improve translation of GWAS results across ancestries. We have revised the manuscript to more clearly communicate this.*

- Are the conclusions adequately supported by the data shown? If not, please explain

No. The PTRS is not shown to outperform the PRS approach for European and Asian groups. And the recently proposed PRS methods should be considered in this comparison.

*Reply: We have revised the paper to more clearly communicate that we are not trying to show that this method performs better than PRS. We do not find evidence that PTRS outperforms portability in European and Asian groups, which is likely due to limited power of our approach indicating the need to improve predictors and better resolved reference transcriptomes.*

- Are sufficient details provided to allow replication and comparison with related analyses that may have been performed? If not, please specify what is required.

Yes.

- Does the work represent a significant advance over previously published studies?

No. See my comments below.

*Reply: Our goal is to provide proof of concept that leveraging transcriptomes can help improve portability across ancestries and that investing in reference transcriptomes should be considered in the effort to increase diversity of GWAS.*

- Is the paper of broad interest to others in the field, or of outstanding interest to a broad audience of biologists?

Yes for the topic of translating GWAS findings across ethnicity groups. No for the proposed approach.

*Reply: We believe that our initial submission was seen as an alternative to existing PRS methods, whereas that was never our intention. We have revised the manuscript to clarify why we think our conclusions are important. Our results emphasize the benefits of informing PRS with likely causal factors as well as the potential benefits of diversifying reference transcriptomes and other omics.*

Even though it is interesting for this paper to derive a polygenic transcriptome risk score (PTRS) to investigate the predictivity of genetically regulated gene expression values for phenotypes and how this PTRS might be portability across different ethnicity groups, I think the recently proposed PRS methods including the PRS-CS method (G. Tian et. al, nature Communications, 2019, https://www.nature.com/articles/s41467-019-09718-5) accounting for LD and XP-BLUP (Coram et al. AJHG 2017, 10.1016/j.ajhg.2017.09.005 ) modeling discovery and test GWAS summary statistics data sets that are of different ethnicities are preferred ways to translate GWAS summary statistics obtained in a discovery data set to another test GWAS summary statistics data set of different ethnicity by deriving polygenic risk scores. Here are my main concerns:

1. Especially, given the subtle difference between the phenotype risk predictivity by PTRS and the standard PRS with respect to "portability" as shown in the paper, I think it is worth to compare with the PRS derived by PRS-CS with tunning parameters determined by the test dataset and the XP-BLUE method.

*Reply: We agree with the reviewer that comparing with other PRS methods is important when proposing a new method. However, our goal here is to provide a proof of concept that using causal mediating traits provide different avenues to improve portability and show the benefits of it. We are not claiming this approach as a replacement of existing PRS methods.*

*Action: we clarify the scope of the manuscript throughout the revised manuscript. For example, we changed the title from “PTRS improves portability of PRS across ancestries” to “PTRS can improve portability of PRS across ancestries” We also provide a version of the manuscript with the changes highlighted.*

2. As shown from the trained gene expression prediction models by PrediXcan with the GTEx reference data, only ~5000 genes would have a valid prediction model per tissue type. The poor performance of accounting <50% SNP heritability by PTRS and not outperforming PRS in test data of European and Asian ethnicities might due to not accounting for causal SNPs whose effects on phenotype are not mediated through transcriptome. This also show the proposed PTRS should not be used alone for predicting phenotype risks.

*Reply: As mentioned above, we agree that PTRS does not out-perform PRS due to the fact that PTRS captures variations mediated through predictable transcriptome. And we agree that PTRS should not be considered a replacement of PRS. Our main message is that using mediating causal traits should be part of the multi pronged strategy to improve PRS portability and ultimately performance.*

3. Since the SNP heritability is known not uniformly distributed over all genome-wide SNPs as shown by all GWAS results, comparing PVE due to predicted gene expression to the "expected" phenotype heritability does not make sense. The P+T standard PRS method might even show a higher fold of difference with respect to the "expected" phenotype heritability.

*Reply: If the reviewer is referring to the 20-fold increase in the the expected proportion of heritability explained in page 6, we have modified the language to “Per feature proportion of heritability explained” We also rephrased the paragraph on the relative performance of PTRS vs PRS as follows: “However, it is worth noting that since predicted transcriptomes were explaining about a fifth of the heritability (i.e. what common genetic variants could explain), we would expect that the prediction performance of PTRS would be about a fifth of the PRS performance. However, PTRS performance was around half of the PRS performance. This better than expected performance suggests that integrating predicted transcriptomes and other omics is a promising avenue to improve PRS performance in general.”*

4. As mentioned by the authors, using the same test data to tune prediction model for both P+T PRS or PTRS is not suitable in practice. One could use half of the test GWAS data as a validation data set to tune these parameters, and then test on the other half of the test GWAS data.

*Reply: We simply followed the procedure adopted by Martin et al (“Relative prediction accuracy” in Supplementary Information) to facilitate comparison.*

*Now we followed the reviewer’s suggestion and split the target set into validation and test set. We calculate the hyperparameters (penalty for elastic net/PTRS and p-value threshold for PRS) in the validation set and calculate the R2 under the selected hyperparameter in the test set. To reduce the stochasticity of the estimated performance we repeat the random splitting 10 times and report the average R2 across the ten splitting schemes. As before, the weights are calculated in the discovery set for different hyperparameters.*

*We have updated all the results with this new testing approach and the substance of the results remain unchanged. The following figure shows the comparison of R2 using the new vs. the old approach.*

*Action: we have edited the results section reflecting this change in the estimation of R2. The new paragraph reads “We split the target set into validation and test set. We calculated the hyperparameters (penalty for elastic net/PTRS and p-value threshold for PRS) in the validation set and calculated the $\pRsq$ under the selected hyperparameter in the test set. To reduce the stochasticity of the estimated performance we repeated the random splitting 10 times and report the average $\pRsq$ across the ten splitting schemes. The weights were calculated in the discovery set for the different hyperparameters.” (page 8).*

Reviewer 2:

This paper by Liang et al. proposes a new method to leverage genetically predicted gene expression levels by integrating them into a polygenic transcriptome risk score (PTRS) that can be used for complex trait prediction. The authors show that the PTRS have slightly weaker predictive ability compared to normal PRS trained using GWAS weights. However, the authors hypothesized the predicted transcriptome information may be more consistent across populations than GWAS-weights and test whether PTRS trained using European transcriptome data may have improved prediction in other ancestries compared to PRS. The authors show that these PTRS are at least as portable as PRS across ancestry groups while being slightly more portable than PRS in African ancestry participants (whose genetic distance is highest from the training population). Overall I think that this is an illustrative study about how gene-expression information may improve portability; however, I outline some concerns that I think can easily be addressed with additional analyses of the data at hand.

Major comments:

-My primary concern with the paper's methodology is that the PRS and PTRS are optimized for each target population (lines 169-71), leaving each target set to have a different set of genes or variants in the model. This is a problem for two reasons: (1) it is dissimilar from traditional PRS analyses the p-value threshold is exclusively learned in the discovery or tuning dataset - the performance (e.g. r2) should only be measured for external validation samples to avoid inflated performance metrics, (2) PRS transferability is usually compared using the single model (e.g. single p-value/LD-threshold) that is applied and evaluated in multiple populations. My interpretation of the current analysis is that it measured the portability of the underlying predictdb data rather than actual scores since this paper uses a target-sample specific p-value threshold (PRS) or lambda value (PTRS) - this is also inconsistent with the terms 'weights from UK Biobank Discovery Set' labels in Figure 1. I think the analysis needs to be redone with a single PRS and PTRS learned in the discovery and EUR ref datasets that are then evaluated as-is in the different ancestry groups to make the specific claims about score portability.

*Reply: We simply followed the procedure adopted by Martin et al (“Relative prediction accuracy” in Supplementary Information) to facilitate comparison. This revised manuscript follows the reviewer #1’s suggestion and split the target set into validation and test set and expect that following reviewer #2’s suggestion will yield similar results. We calculate the hyperparameters (penalty for elastic net/PTRS and p-value threshold for PRS) in the validation set and calculate the R2 under the selected hyperparameter in the test set. To reduce the stochasticity of the estimated performance we repeat the random splitting 10 times and report the average R2 across the ten splitting schemes. As before, the weights are calculated in the discovery set for different hyperparameters.*

*We have updated all the results with this new testing approach and the substance of the results remains unchanged. The following figure shows the comparison of R2 using the new vs. the old approach.*

*Action: we have edited the results section reflecting this change in the estimation of R2. The new paragraph reads “We split the target set into validation and test set. We calculated the hyperparameters (penalty for elastic net/PTRS and p-value threshold for PRS) in the validation set and calculated the $\pRsq$ under the selected hyperparameter in the test set. To reduce the stochasticity of the estimated performance we repeated the random splitting 10 times and report the average $\pRsq$ across the ten splitting schemes. The weights are calculated in the discovery set for the different hyperparameters.” (page 8).*

-The current display of the data in Figure 4A may be misleading to some readers, as it shows the predictive accuracy of the EUR ref PTRS as equivalent to the EUR ref PRS. The figure legend and text should be clarified to reflect this, as it doesn't accurately display that the PTRS underperforms the PRS across ancestries. Perhaps including a version of Figure 3A comparing PRS vs. PTRS using the GTex EUR data in all target datasets might adequately fulfill this criterion, such that you see the absolute comparison of PRS/PTRS in each ancestry as well as the relative performances in the current figure 4a. In addition, I think Figure 4B should be extended to all target ancestries so that readers can see if the GTex result is consistent across all ancestries using MESA EUR data as the improved portability claim is only made using p-values of t-tests in the African target sample (tail of the genetic distance distribution) rather than using some sort of trend-test of the decline across strata.

*Reply: We have clarified in the caption that the actual performance measures are available in Fig 3A and in the supplement.*

*We have added supplementary figure S3 with performance for all ancestries.*

-What is the rationale behind not using the MESA ALL ancestry data as a source of data? Multi-ancestry GWAS SumStats usually improve cross-population portability, it would be interesting to see if the same can be said of multi-ancestry genetically-predicted transcriptomes.

*Reply: To make the comparison fair, we wanted to use models which were trained with similar sample sizes. MESA AFHI and MESA EUR happened to be based on the same sample size (585 vs. 578). We are in the process of generating trans-ancestry models with the newer data from MESA/TOPMED and we will address this point with this higher quality dataset in the near future.*

-The sample sizes of the non-European ancestry groups in UK Biobank are very small compared to previous analyses of the same data. Specifically, the number of African individuals is considerably smaller than seen in other papers (it seems you've excluded self-reported Caribbean identity), and the South Asian sample seems to have excluded British Pakastani and Bangladeshis? I'd suggest revisiting the inclusion criteria and rerunning the clustering to increase the sample size.

*Reply: We followed the reviewer’s suggestion. We added Pakistani and Bangladeshi as part of S.ASN samples. And we also included Caribbean samples as part of the analysis. Some of the results with the MESA predictors lost significance with the expanded sets so they have been updated and the conclusions have been revisited. Our key result that PTRS was more transferable than PRS was robust to these changes.*

Minor comments:

-I found the data about which transcriptome is being predicted a little confusing throughout the manuscript. It is initially introduced as whole blood, but then Figure2A shows that including more Gtext tissues improves the PVE. But then the rest of the paper uses MESA and doesn't say the tissue. I wonder if the multi-tissue result should be presented as a final result/discussion point to motivate methodological developments and new data?

*Reply: Thanks for the suggestions. We edited the manuscript to clarify that we use GTEx whole blood and MESA monocyte data. Action: We added to the caption of Figure 2A that “Notice that in general we used GTEx whole blood and MESA monocyte-based predictors except for this panel where we used 10 tissues from GTEx.” (caption Figure 2) We added some discussion about the multi-tissue PTRS in the discussion section. “The increase in the proportion of variance explained from a fifth when using whole blood predictors alone compared to a third when using 10 tissues indicates that much can be gained by increasing the breadth of reference omic data.” (page 13)*

-How different are the genes selected in the PTRS across different ancestry groups and source datasets?

*Reply: We generated a Venn diagram comparing the gene sets being used in the best-performing PTRS of each ancestry group. See results here: https://drive.google.com/file/d/1Dn6tdmMZNFk-XGckyD1wFj_0oNCyP cjM We observed quite a bit of heterogeneous patterns across different traits. For some traits, such as basophil, the best performing model of AFR contains the fewest number of genes. Whereas, for some traits like BMI, the selected genes are mostly shared. For some traits like mean corpuscular volume, it seems that there are a set of genes being selected only in EUR, AFR, and S.ASN but not E.ASN.*

-It would be nice to have some mention of this paper's relationship to the related Gene Expression Risk Score method (Pain et al. 2021 doi:10.1093/hmg/ddab053).

*Reply: Added to the introduction that As shown by \citep{Pain2021-iv}, PTRS alone does not outperform the state of the art PRS.*

-I wonder if the authors have access to disease phenotypes (CAD/Stroke/Diabetes)? How does the PTRS data perform at predicting binary phenotypes?

*Reply: In general, for relatively balanced studies, we found the quantitative approximation to binary traits to work well. We will investigate the performance differences in future work.*

-Is portability the best word to describe performance differences across ancestries? Generalizability or transferability is more commonly seen in the polygenic score literature.

*Reply: We believe that portability is a term that is being also adopted to mean generalizability or transferability. See for example, Amariuta et al’s “Improving the trans-ancestry portability of polygenic risk scores by prioritizing variants in predicted cell-type-specific regulatory elements”.*

-The mention of cross-species trait prediction in the abstract seems unrelated to the current research. I suggest limiting this to the discussion, however, I think the use of PTRS in this scenario is still unrelated to transferability across populations.

*Reply: We have modified the cross species comments to tie it better with the discussion of this paper.*

Reviewer 3:

This work proposes the development and use of a transcriptomic risk score (PTRS) model for trans-ethnic risk prediction. Rather than using SNPs to build a predictive scores (as in a PRS) the PTRS uses predicted expression to build a predictive score. The work investigates multiple aspects of the PTRS model. The PTRS features are shown to be substantially enriched for heritability. Perhaps the key result is that, although the PTRS has worse overall performance than the PRS, it carries over *relatively* better into non-European populations.

The manuscript is well written, tackles an interesting problem in a creative way, and is thorough. My main concerns are about the robustness of the primary result about PTRS portability. Given that the PTRS itself is not as accurate as the PRS, it is critical to show definitively that PTRS is more portable and understand why.

*Reply: Thank you for the excellent summary of our paper :).*

\# Major comments

* My main concern is the very modest improved portability of the PTRS vs PRS. In particular, the gain observed from GTEx EUR data into AFR (p=0.03) does not appear to replicate when using MESA EUR data (p=0.13) (Figure 4). Can the authors comment this lack of reproducibility? In Figure 4, some of the PTRS improvements appear to be outliers (i.e. a long tail of points with high y-values), are these outliers driving the significant difference? For example, does winsorizing the portability metrics retain the significant difference in portability? Given that portability is defined as a ratio, is it possible that traits with very low predictive accuracy are producing outsized portability ratios and adding more noise than signal? Is there anything interesting about individual traits that port over much better by PTRS than by PRS?

*Reply: We believe that the MESA based prediction models are not as reliable as the GTEx models since they are based older array based technology.*

*We followed the reviewer’s suggestions and added the portability vs performance figures to the supplement (supplementary figure 4).*

*We used wilcoxon rank test and found that the increased portability of PTRS relative to PRS in African target set remained significant. Also with the addition of Caribbeans (as requested by reviewer #2), the new results do not have an obvious outlier.*

*Action: we now report the wilcoxon test p-value instead of the paired t-test.*

* The Discussion notes that the PTRS was trained using individual-level data and the PRS using summary-level data. It is also important to show that the improved portability is not simply a case of using a better training model for the PTRS. This could be addressed by retraining the PRS using individual-level data or retraining the PTRS using summary-level data to make this analysis consistent. Relatedly, the Results note that some in-sample parameter tuning was performed on the two scores (which is common), can the improved portability of the PTRS be an artifact of the in-sample tuning (i.e. the PTRS is a bit more overfit)? This could be addressed by performing a double cross-validation where the final target data is completely held out; or by using a prediction scheme that does not require in-sample parameter tuning.

*Reply: For a more fair comparison we calculated PTRS using summary statistics and p-value thresholding method, as used for PRS.*

*As expected this approach yielded, on average, lower R2 than the elastic net approach.*

*However, the new results did not affect our conclusions. In other words, the improved portability was not due to better prediction models for PTRS. Action: we have added the comparison to the supplementary figure 1. We clarified in the text that using elastic net vs summary statistics with p-value thresholding was not the reason for the increased portability of PTRS by adding the following sentence to the results section. “We verified that the increase in portability of PTRS in the African target set was not driven by the use of elastic net instead of p-value thresholding for building the PTRS as shown in supplementary figure 2 where portability is shown for the p-value thresholding approach.” (page 11).*

* I'm puzzled by the proposed use case for this approach. Since one will always be able to generate a PRS if one has a PTRS, is there ever a case where improvement of the PTRS in isolation is meaningful? Isn't the metric of interest whether PRS+PTRS improves over PRS and/or improves in portability over PRS? Does PRS+PTRS do better in terms of portability than PRS alone? If PRS+PTRS is not better than PRS does this result have any practical application or is it more a biological/statistical curiosity?

*Reply: Optimal strategies to improve PRS using PTRS will be the natural follow up to our study. The main goal of this paper is to demonstrate the benefits of adding transcriptome regulation to the construction of PRS.*

\# Minor comments

* Figure 1 is very helpful!

* I'm puzzled by the use of LDSC regression to estimate SNP-heritability rather than using the exact variance components approach that was used to estimate T-heritability. From what I understand, LDSC regression is intended for estimating partitioned heritability and does not produce unbiased estimates of total heritability. Alternatively, it seems like the authors could have estimated the T-heritability using LDSC regression to make the apples-to-apples comparison.

*Reply: We calculated h2 using REML and found that they are concordant with LDSC approach.*

*We also updated the fraction of heritability being explained by PTRS PVE since the denominator changed.*

* line 131: "This result is largely consistent to the estimates reported previously using a different approach" -- is this missing a figure/table?

*Reply: A reference to Yao et al was missing. Action: we edited the sentence as follows “This result is largely consistent to the estimates reported previously by \citep{yao:2019} for the subset of traits used here which are mostly blood related.”*

* line 155: "For the European target set, the difference between using the MESA AFHI or the EUR transcriptomes was not significantly different from". This is interesting and surprising, can the authors comment on the asymmetry?

*Reply: We are not sure how to interpret this asymmetry but we are currently investigating this issue with better non-European reference transcriptome data in TOPMED. In this revision, we removed the focus on the old array based models since they were not robust to different approaches suggested by the reviewers.*

* line 175: These results seem consistent with T features capturing more heritability *per feature* than SNP features, and thus having less noise in the final score. There is a known relationship between heritability, GWAS sample size, number of SNPs, and expected prediction R^2 (for example: PMID 18852893). Can the authors comment on whether the R^2 they get from the PRS and PTRS are consistent with these expectations? This would help guide extrapolation to other PTRS studies.

*Reply: We thank the reviewer for pointing us to this interesting fact. We agree that our better than expected performance of PTRS is related to the per feature heritability explained by predicted genes and Daetwyler et al’s formula. In the following figure (now it is Fig3B) we observe that PTRS is much closer to the optimal R2 given by the PVE than PRS, consistent with an increase in performance when the per feature heritability is higher.*

*Action: We added this figure as Fig3B and the following paragraph to the results section. “We found that PTRS was much closer to the optimal performance upper bound (PVE) than PRS was to its upper bound (heritability), as shown in Fig.~\myref{fig:fig3}B where each score is compared to its upper bound. This is consistent with the reported monotonically increasing relationship between a measure of prediction performance (correlation between the genetic component and the predicted values, slightly different from our definition, using lowercase $r$ to distinguish) and the per-feature heritability/PVE: $r^2 = \frac{n \cdot h^2/n_\text{features}}{ 1 + n \cdot h^2/n_\text{features}}$ \cite{Daetwyler2008-wq}. The higher than expected prediction performance of PTRS indicates that the predicted expression of each gene carries a higher per feature PVE than genetic variant’s per feature heritability.”*

**Second round of review**

**Reviewer 1**

The authors emphasized in the response letter that this manuscript is not aim to propose a new PRS method but to show the improved portability of the PTRS than PRS. For this purpose, it is essential to show how PTRS can actually improve our understanding of the complex phenotype, such as improve the risk prediction accuracy when included as an additional predictor of PRS. Otherwise, what would be the practical meaning or usefulness of this PTRS score?

**Reviewer 2**

Overall, I think the rigor of the analysis has improved from the initial submission with respect to the model building (proper hold-outs), and increasing the sample sizes of the African and South Asian ancestry groups in the study. However, I still have some outstanding concerns with the revised manuscript that I outline below:

Major Comments:

- I think the removal of the MESA-based prediction models from Figure 4B weakens the paper by omitting results that don’t meet the threshold for statistical significance but did support the pattern of improved portability from PTRS and ancestry-matching. I worry that these data have been omitted because the improvements seen in the lower left-hand of the Target: UKB AFR graph in Figure 3C are reduced in the current version of the manuscript (which to me highlights that they may have been artifacts of the evaluation dataset or re-optimized scores from the previous iteration of the analysis). My previous comment was that the analysis should have been expanded to all target sets using both the MESA EUR and MESA AFHI models, given that these datasets are of equivalent size to the Gtex Whole_blood cohort I would request that the expanded analysis be done and re-included in the manuscript.

- I find the authors justification of ensuring comparable training sample-sizes to be an insufficient reason for excluding the multi-ancestry MESA data. If the point of the manuscript is to illustrate how PTRS models can improve portability they shouldn’t leave out a possible improvement based on multi-ancestry models which are already available to them. I believe that a positive result using this method would compensate for the lack of performance of the single-ancestry MESA models and not compromise any future manuscripts that they wish to write using new models derived from RNA-seq.

- The colours of the points in Figures 2B and 3C are never explained.

Minor Comments: - Abstract and elsewhere you refer to “African samples” or “non-European-descent” but these individuals are from the UK biobank. I think a careful edit of the manuscript is warranted to clarify that you are talking about “individuals of African ancestry” or “European ancestry” as you’re speaking about genetic ancestry and not samples.

- Line 45 “… now widely understood that most association signals are driven by their effects on the transcriptome”. I’m not sure this is true as some signals could be for protein expression, splicing, isoforms, etc. ”. I think this requires citation or toning down with ‘many’.

- Caption of Figure 2: “In the European set, the difference between the two transcriptomes is not significant. The vertical bars show the 95% confidence intervals estimated with paired t-test.” – but there don’t appear to be any confidence intervals.

**Reviewer 3**

The authors have addressed most of my major and minor concerns but my major comment #3 remains outstanding. The response states "The main goal of this paper is to demonstrate the benefits of adding transcriptome regulation to the construction of PRS" however it is not clear that the results in the revision demonstrate the benefits of adding PTRS to the construction of PRS, rather they demonstrate the improved portability of PTRS (on it's own) relative to the portability of PRS (on it's own). As far as I can tell an analysis of adding PTRS to the construction PRS is not presented at all. I previously recommended resolving this question directly (prev major comment #3: "Does PRS+PTRS do better in terms of portability than PRS alone? If PRS+PTRS is not better than PRS does this result have any practical application or is it more a biological/statistical curiosity?") but the authors have elected not to address this even though the analysis would simply require adding the PTRS as a feature in the PRS or putting the PTRS + PRS into a joint model in the target non-European data and reporting the adjusted R^2 and portability. Currently, there are multiple statements in the paper that could be interpreted to mean PRS+PTRS improves portability over PRS when all that has been shown is that PTRS is more portable but less predictive than PRS, including:

* The Title: "PTRS can improve portability of PRS" * The Introduction: "we hypothesized that prediction at the level of estimated transcript abundance rather than SNPs might help the portability across populations"

* The Section Title: "PTRS improves portability into the African population"

* The Results: "These results provide strong proof of principle that integrating predicted transcriptome as done with PTRS has the potential to improve portability of risk scores across populations"

* The Discussion: "Our study results suggest that investing in multi-omic studies of diverse populations may be an effective way to reduce current genomic disparities by taking better advantage of existing GWAS studies." If PTRS+PRS does not significantly outperform PRS alone in the African cohort it remains unclear to me what potential the PTRS holds for reducing disparities, and these statements should be more clearly presented as speculative.

**Authors Response**

**Point-by-point responses to the reviewers’ comments:**

Reviewer 1:

The authors emphasized in the response letter that this manuscript is not aim to propose a new PRS method but to show the improved portability of the PTRS than PRS. For this purpose, it is essential to show how PTRS can actually improve our understanding of the complex phenotype, such as improve the risk prediction accuracy when included as an additional predictor of PRS. Otherwise, what would be the practical meaning or usefulness of this PTRS score?

*Reply: We have addressed this comment in the current revision by demonstrating that combining PTRS with PRS yields higher prediction performance and portability into AFR as shown in the new figure 5 shown below. In particular, note in Fig. 5c that we observed substantial evidence of improved portability when we combined the PTRS with the PRS (P=0.0021).*

*To rule out that the improvement in performance was due to the better prediction approach used for PTRS (elastic net on individual level data instead of summary statistics based clumping and thresholding), we also performed the previous analysis using summary statistics clumping and thresholding PTRS and found that the direction of the gain in prediction performance was maintained although the significance was lost. The gain in portability in the AFR population remained significant (p=0.011).*

Reviewer 2:

Overall, I think the rigor of the analysis has improved from the initial submission with respect to the model building (proper hold-outs), and increasing the sample sizes of the African and South Asian ancestry groups in the study. However, I still have some outstanding concerns with the revised manuscript that I outline below:

Major Comments:

- I think the removal of the MESA-based prediction models from Figure 4B weakens the paper by omitting results that don’t meet the threshold for statistical significance but did support the pattern of improved portability from PTRS and ancestry-matching. I worry that these data have been omitted because the improvements seen in the lower left-hand of the Target: UKB AFR graph in Figure 3C are reduced in the current version of the manuscript (which to me highlights that they may have been artifacts of the evaluation dataset or re-optimized scores from the previous iteration of the analysis). My previous comment was that the analysis should have been expanded to all target sets using both the MESA EUR and MESA AFHI models, given that these datasets are of equivalent size to the Gtex Whole_blood cohort I would request that the expanded analysis be done and re-included in the manuscript.

*Reply: Here are the results of MESA-based prediction models on all the populations. We note that the median portability of MESA AFHI models in EUR test is lower than 1 and it is expected since the portability is calculated relative to the MESA EUR models in EUR ref.. Below we show the zoom-in plot focusing on AFR only.*

*When we added the Caribbeans to our analysis, the expected trend of improved portability as the training ancestry got closer to the AFR population was lost. We thought that given the obvious limitations of using an older array technology, it would be better to drop the results but we will add it to the supplement clarifying that the trend we found in the earlier version of the manuscript did not hold and that the analysis should be revisited in the future with better predictors.*

- I find the authors justification of ensuring comparable training sample-sizes to be an insufficient reason for excluding the multi-ancestry MESA data. If the point of the manuscript is to illustrate how PTRS models can improve portability they shouldn’t leave out a possible improvement based on multi-ancestry models which are already available to them. I believe that a positive result using this method would compensate for the lack of performance of the single-ancestry MESA models and not compromise any future manuscripts that they wish to write using new models derived from RNA-seq.

*Reply: We included the results from MESA ALL models in the above figure.*

- The colours of the points in Figures 2B and 3C are never explained.

*Reply: These colors indicate the type of phenotypes. We drop the coloring to simplify the presentation. Updated Figure 2B.*

Minor Comments:

- Abstract and elsewhere you refer to “African samples” or “non-European-descent” but these individuals are from the UK biobank. I think a careful edit of the manuscript is warranted to clarify that you are talking about “individuals of African ancestry” or “European ancestry” as you’re speaking about genetic ancestry and not samples.

*Reply: We made the changes accordingly as seen in the track change version of the manuscript.*

- Line 45 “… now widely understood that most association signals are driven by their effects on the transcriptome”. I’m not sure this is true as some signals could be for protein expression, splicing, isoforms, etc. ”. I think this requires citation or toning down with ‘many’.

*Reply: We followed the reviewer’s suggestion changing “most” to “many”.*

- Caption of Figure 2: “In the European set, the difference between the two transcriptomes is not significant. The vertical bars show the 95% confidence intervals estimated with paired t-test.” – but there don’t appear to be any confidence intervals.

*Reply: Thanks for catching this error. We updated the figure but not the caption. We fixed this issue.*

Reviewer 3:

The authors have addressed most of my major and minor concerns but my major comment #3 remains outstanding. The response states "The main goal of this paper is to demonstrate the benefits of adding transcriptome regulation to the construction of PRS" however it is not clear that the results in the revision demonstrate the benefits of adding PTRS to the construction of PRS, rather they demonstrate the improved portability of PTRS (on it's own) relative to the portability of PRS (on it's own). As far as I can tell an analysis of adding PTRS to the construction PRS is not presented at all. I previously recommended resolving this question directly (prev major comment #3: "Does PRS+PTRS do better in terms of portability than PRS alone? If PRS+PTRS is not better than PRS does this result have any practical application or is it more a biological/statistical curiosity?") but the authors have elected not to address this even though the analysis would simply require adding the PTRS as a feature in the PRS or putting the PTRS + PRS into a joint model in the target non-European data and reporting the adjusted R^2 and portability. Currently, there are multiple statements in the paper that could be interpreted to mean PRS+PTRS improves portability over PRS when all that has been shown is that PTRS is more portable but less predictive than PRS, including:

* The Title: "PTRS can improve portability of PRS" * The Introduction: "we hypothesized that prediction at the level of estimated transcript abundance rather than SNPs might help the portability across populations"

* The Section Title: "PTRS improves portability into the African population"

* The Results: "These results provide strong proof of principle that integrating predicted transcriptome as done with PTRS has the potential to improve portability of risk scores across populations"

* The Discussion: "Our study results suggest that investing in multi-omic studies of diverse populations may be an effective way to reduce current genomic disparities by taking better advantage of existing GWAS studies."

If PTRS+PRS does not significantly outperform PRS alone in the African cohort it remains unclear to me what potential the PTRS holds for reducing disparities, and these statements should be more clearly presented as speculative.

*Reply: We have addressed this comment in the current revision by demonstrating that combining PTRS with PRS yields higher prediction performance and portability into AFR as shown in the new figure 5 shown below. In particular, note in Fig. 5c that we observed substantial evidence of improved portability when we combined the PTRS with the PRS (P=0.0021).*

*To rule out that the improvement in performance was due to the better prediction approach used for PTRS (elastic net on individual level data instead of summary statistics based clumping and thresholding), we also performed the previous analysis using summary statistics clumping and thresholding PTRS and found that the direction of the gain in prediction performance was maintained although the significance was lost. The gain in portability in the AFR population remained significant (p=0.011).*

**Third round of review**

**Reviewer 1**

The authors have addressed my previous major comment about showing the improvement by using PRS + PTRS. The main message I read from the manuscript is that PRS + PTRS would provide the most "portable" risk score with the highest prediction accuracy for phenotype. I think this main message shall be emphasized in the Abstract/Introduction/Discussion.

In the current manuscript, I found there are many statements in the manuscript that are not quite rigorous and led reader to directions that the authors said they were not trying to address.

Mainly,

1. In the beginning of the Introduction, the authors said "Polygenic risk scores (PRS) for a variety of traits are becoming accurate enough to be useful for clinical practice, realizing the longstanding goal of personalized medicine." I think current studies all show that PRS is useful but still not accurate enough to be used in clinical practice, and PRS is definitely not yet realizing the goal of personalized medicine.

2. In the second paragraph of Introduction section, the authors said "Therefore, methods that can use GWAS results from one population for prediction in other ancestry groups are highly desirable." That makes readers looking forward to a methodology to leverage GWAS results across populations. As mentioned in my comments for the initial submission, there are many such methods available. But the authors said "they are not trying to address this research problem by PTRS" in their response letter of R1. If so, such strong statement sentences shall be rewritten to be consistent with the results shown in this manuscript.

3. The authors shall also discuss their "predicted expression levels" are only using cis-SNPs. There would be a proportion of heritability explained by trans-SNPs missing in their current approach.

4. "PTRS' prediction accuracy approaches the upper bound given by their PVE". The way the authors define the upper bound of PVE is linearly proportion to the number of SNPs used depends on the assumption that all SNPs explain PVE evenly. However, we know this is not true by showing SNPs have all different GWAS p-values. That is, the proportion of PVE explained by a random collection of 1K SNPs would be much smaller than the proportion explained by the top significant 1K SNPs. I think the statements in this sub-section need to be re-written, or a more realistic and rigorous "upper bound" need to be statistically derived.

5. There are plenty of question marks that should be referred to supplement figure or table numbers. I did not find a supplement file of supplemental figures in the submission. The collection of supplement files contain *.aux files that are confusing.

**Reviewer 2**

The authors have addressed my concerns. I think the paper illustrates the method and results well; however I think more data or methodological improvements are still required to unequivocally show that PTRS improve portability (especially because this result is limited to the African ancestry subset of UK Biobank). Perhaps this could be solved with the multi-tissue data as is mentioned in the discussion.

**Reviewer 3**

The authors have addressed my concerns. One minor presentation point (which does not need re-review) is that it's now a bit difficult to identify the relevant per-trait results from the Supplementary Tables, it would be helpful to have one additional Supplementary table with the per-trait PRS, PTRS, and PRS+PTRS accuracies in the test set side-by-side for easier comparison.

**Authors Response**

**Point-by-point responses to the reviewers’ comments:**

*Reply: We've addressed these comments in the manuscript.*
